# Introgression of clubroot resistant gene into ***Brassica oleracea*** L. from ***Brassica rapa*** based on homoeologous exchange

**DOI:** 10.1093/hr/uhac195

**Published:** 2022-08-30

**Authors:** Mingzhao Zhu, Limei Yang, Yangyong Zhang, Mu Zhuang, Jialei Ji, Xilin Hou, Zhansheng Li, Fengqing Han, Zhiyuan Fang, Honghao Lv, Yong Wang

**Affiliations:** Key Laboratory of Biology and Genetic Improvement of Horticultural Crops, Ministry of Agriculture, Institute of Vegetables and Flowers, Chinese Academy of Agricultural Sciences, Beijing 100081, China; Institute of Vegetable Science, Beijing Academy of Agricultural and Forestry Sciences, Beijing 100097, China; State Key Laboratory of Crop Genetics and Germplasm Enhancement, College of Horticulture, Nanjing Agricultural University, Nanjing, Jiangsu 210095, China; Key Laboratory of Biology and Genetic Improvement of Horticultural Crops, Ministry of Agriculture, Institute of Vegetables and Flowers, Chinese Academy of Agricultural Sciences, Beijing 100081, China; Key Laboratory of Biology and Genetic Improvement of Horticultural Crops, Ministry of Agriculture, Institute of Vegetables and Flowers, Chinese Academy of Agricultural Sciences, Beijing 100081, China; Key Laboratory of Biology and Genetic Improvement of Horticultural Crops, Ministry of Agriculture, Institute of Vegetables and Flowers, Chinese Academy of Agricultural Sciences, Beijing 100081, China; Key Laboratory of Biology and Genetic Improvement of Horticultural Crops, Ministry of Agriculture, Institute of Vegetables and Flowers, Chinese Academy of Agricultural Sciences, Beijing 100081, China; State Key Laboratory of Crop Genetics and Germplasm Enhancement, College of Horticulture, Nanjing Agricultural University, Nanjing, Jiangsu 210095, China; Key Laboratory of Biology and Genetic Improvement of Horticultural Crops, Ministry of Agriculture, Institute of Vegetables and Flowers, Chinese Academy of Agricultural Sciences, Beijing 100081, China; Key Laboratory of Biology and Genetic Improvement of Horticultural Crops, Ministry of Agriculture, Institute of Vegetables and Flowers, Chinese Academy of Agricultural Sciences, Beijing 100081, China; Key Laboratory of Biology and Genetic Improvement of Horticultural Crops, Ministry of Agriculture, Institute of Vegetables and Flowers, Chinese Academy of Agricultural Sciences, Beijing 100081, China; Key Laboratory of Biology and Genetic Improvement of Horticultural Crops, Ministry of Agriculture, Institute of Vegetables and Flowers, Chinese Academy of Agricultural Sciences, Beijing 100081, China; Key Laboratory of Biology and Genetic Improvement of Horticultural Crops, Ministry of Agriculture, Institute of Vegetables and Flowers, Chinese Academy of Agricultural Sciences, Beijing 100081, China

## Abstract

Clubroot is a soil-borne disease in cabbage (*Brassica oleracea* L. var. *capitata* L.) caused by *Plasmodiophora brassicae*, which poses a great threat to cabbage production. However, clubroot resistance (CR) genes in *Brassica rapa* could be introduced into the cabbage via breeding to make it clubroot resistant. In this study, CR genes from *B. rapa* were introduced into the cabbage genome and the mechanism of gene introgression was explored. Two methods were used to create CR materials: (i) The fertility of CR Ogura CMS cabbage germplasms containing *CRa* was restored by using an Ogura CMS restorer. After cytoplasmic replacement and microspore culture, *CRa*-positive microspore individuals were obtained. (ii) Distant hybridization was performed between cabbage and *B. rapa,* which contained three CR genes (*CRa, CRb*, and *Pb8.1*). Finally, BC_2_ individuals containing all three CR genes were obtained. Inoculation results showed that both *CRa*-positive microspore individuals and BC_2_ individuals containing three CR genes were resistant to race 4 of *P. brassicae*. Sequencing results from *CRa*-positive microspore individuals with specific molecular markers and genome-wide association study (GWAS) showed penetration at the homologous position of the cabbage genome by a 3.42 Mb *CRa* containing a fragment from *B. rapa*; indicating homoeologous exchange (HE) as the theoretical basis for the introgression of CR resistance. The successful introduction of CR into the cabbage genome in the present study can provide useful clues for creating introgression lines within other species of interest.

## Introduction

*Brassica* is one of the most important genera in the Cruciferae. The well-known U’s triangle theory reveals that *Brassica* consists of three basic species including *Brassica rapa* (AA, 2n = 20), *Brassica nigra* (BB, 2n = 16) and *Brassica oleracea* (CC, 2n = 18) as well as three composite species including *Brassica napus* (AACC, 2n = 38), *Brassica carinata* (BBCC, 2n = 34) and *Brassica juncea* (AABB, 2n = 36), which naturally doubled from the basic species [1]. The three basic genomes are closely related and contain abundant genetic resources, which provide the foundation for distant hybridization or other methods of breeding in the *Brassica* crops.

The importance of the cultivation of cabbage (*B. oleracea* L. var. *capitata* L.), a member of the U’s triangle, is demonstrated by its economic and nutritional value. In 2019, the production of cabbage and other *Brassica* crops exceeded 70 million tons (http://faostat.fao.org/). However, an increasingly rampant disease (clubroot), caused by *Plasmodiophora brassicae*, posed a huge threat to cruciferous crops [2]. Diseased plants exhibit swollen roots, difficulty absorbing nutrients and water, reduced growth, and eventual wilting or death [3]. Moreover, the resting spores of *P. brassicae* can maintain vitality in the soil for more than 20 years [4] and reinfect new hosts under suitable environmental conditions, making the infected area unsuitable for future growth. Over the past decades, methods developed to control clubroot – such as scientific rotation, adjustment of soil pH and the application of antagonistic microorganisms and germicidal agents – did not demonstrate sufficient control of the disease. Previous study has demonstrated that cultivating clubroot resistance (CR) cabbage varieties is the most effective strategy to control the clubroot due to its environmental friendliness and low cost [5]. However, the lack of resistance materials, complicated genetic mechanisms of resistance, and few available molecular markers make CR breeding of cabbage challenging [6–9]. In recent years, although several QTLs such as *CRQTL-GN_1* [10], *Rcr7* [11], *PCR.II-2* [12], *PbC4.1* [13], and *qCRc7–1* [14] have been mapped in cabbage, no CR gene in cabbage has been cloned so far, which means that it was premature to use the CR genes in cabbage for CR breeding. In contrast, a series of important milestones have been achieved in the CR breeding of *B. rapa*, owing to the advantages of their quality traits or the high contribution rate of quantitative trait loci (QTLs). So far, many CR loci have been identified in *B. rapa*, *B. nigra* and turnip, including *CRa* [15, 16], *CRb* [17], *CRc* [18], *CRd* [5], *CRk* [18], *CRq* [19], *CRs* [20], *Crr1a* [21], *Crr2* [22], *Crr3* [23], *Crr4* [22], *CrrA5* [24], *Rcr1* [25], *Rcr2* [26], *Rcr3* [27], *Rcr6* [28], *PbBa8.1* [29], *BraA.Pb.8.4* [30], *BraA.CR.a* [31], and *BraA.CR.b* [31]*.* These studies have promoted the understanding of the molecular mechanism of CR, and the development of molecular markers that are closely linked to CR genes have accelerated the breeding of CR varieties. Given these results, it may be a feasible strategy to introduce the CR genes from *B. rapa* into the cabbage genome to induce CR.

Distant hybridization can break the boundaries between species and transfer genes mutually, and also introduce new biological characteristics into a species to expand the range of genetic variation, thereby creating new germplasm resources [32]. Distant hybridization can be realized through conventional sexual hybridization or somatic hybridization. Normally, due to the large differences in the number of chromosomes, physiology and biochemistry of the parents, reproductive obstacles such as incompatibility of hybridization and hybrid abortion will occur during sexual hybridization. Embryo rescue and chromosome doubling techniques can effectively overcome the above-mentioned problems, which were widely used in the process of distant hybridization [33]. As the intermediate product of hybridization between *B. rapa* and *B. oleracea*, synthetic *B. napus* has rich structural variation, which was considered to have the potential to cultivate improved varieties [34]. The meiotic chromosome pairing that occurs between homoeologous chromosomes which share a high degree of sequence identity leads to increased homoeologous exchanges (HEs) and gene conversion events [35, 36]. HEs are now known to be common in synthetic polyploids, not only in *B. napus* [37]. This phenomenon may be generalizable across most newly formed polyploids as a result of meiotic instability [38]. Stein *et al.* [39] have confirmed that HE underlies the trait variation in a doubled haploid (DH) population involving a synthetic *B. napus* trait donor and succeeded in narrowing a QTL influencing seed quality traits to a small defined interval, the study of which provided a theoretical basis to a certain extent for the application of HE in breeding.

Although popular CR varieties of cabbage do exist at market, these varieties are sterile. Restoring the fertility of these commercial species is a tough challenge faced by the breeders. Ren *et al.* [40] first reported that Ogura cytoplasmic male sterile (CMS) restorer was used to restore the fertility of Ogura CMS CR cabbages, which carried CR gene *CRb2*. Subsequently, Li *et al.* [41] obtained a restorer cabbage line by transferring the fertility restorer gene *Rfo^B^* into the Ogura CMS line, which was then used to restore the fertility of CR commercial species combined with marker-assisted selection (MAS). The above methods have greatly accelerated the process of CR breeding in cabbage, but it is difficult to obtain restorer lines and the genetic background of the restored progenies is usually complicated, which is not suitable for immediate application in commercial breeding.

Microspore culture, a widely used method in crop breeding, provides an effective technique to obtain a large number of highly homozygous DH parental lines in a short time, which greatly accelerates the breeding process [42, 43]. Additionally, DH populations are also considered as ideal materials for inheritance analysis, linkage map construction and gene mapping [44]. At present, a microspore culture system has been successfully established in rice [45], wheat [46], eggplant [47], and apple [48]. For cabbage, although the system has also been established and applied to disease resistance breeding and QTL mapping [49, 50], there are few reports on the application of microspore culture to cabbage CR breeding [51].

Although *CRa* has been widely used in cabbage resistance breeding, most CR varieties containing *CRa* or other CR genes were monopolized by foreign countries. In China, we urgently need to cultivate CR cabbage varieties with independent intellectual property rights. Therefore, the objective of the current study was to rapidly cultivate CR cabbage materials containing CR genes, by using the cabbage restorer lines and the method of distant hybridization, and to analyse the mechanism of exogenous fragments introgression into cabbage during the distant hybridization process.

## Results

### Fertility restoration of Ogura CMS CR cabbage

In a previous study, we have identified five Ogura CMS (Fig. S1, see online supplementary material) CR cabbage varieties (‘17CR1’, ‘17CR2’, ‘17CR3’, ‘17CR4’, and ‘17CR5’) that were resistant to Pb4 collected from Wulong, Chongqing Municipality, China. The result of the molecular identification marker showed that four (‘17CR1’, ‘17CR2’, ‘17CR3’, and ‘17CR5’) of them contained *CRa*. To restore their fertility, the five varieties were respectively crossed with ‘17Q8–514’, and their offspring were identified by molecular marker Rfo-11 and KBrH129J18. As shown in Table 1, we obtained 1103, 2435, 6021, and 5026 seeds from cross combination of ‘18CR1’, ‘18CR2’, ‘18CR3’, and ‘18CR5’, respectively, and a portion of them was used for screening *Rfo* and *CRa* positive individuals. The results revealed that 15 individuals in ‘18CR3’ and ‘18CR5’ contained *Rfo* (Fig. S2, see online supplementary material), only seven individuals of which contained both *Rfo* and *CRa*, but neither *Rfo* nor *CRa*-positive individuals were observed in both ‘18CR1’ and ‘18CR2’. Results from flowering characters showed that the fertility of ‘18CR3’ and ‘18CR5’ has been restored (Fig. S3, see online supplementary material). Given this, the cabbage inbred lines ‘18Q379’ and ‘18Q887’ as females crossed with ‘18CR3’ and ‘18CR5’ to replace the Ogura CMS cytoplasm. *CRa* marker KBrH129J18 was detected and three *CRa*-positive individuals with relatively fine genetic backgrounds from each combination were selected and planted in a greenhouse for subsequent experiment.

**Table 1:** Results of identification of offspring from four cross combinations between four CR Ogura CMS cabbages and ‘17Q8–514’.

Serial number of cross combination	Number of seeds	Number of applied plants	Number of *Rfo* positive individuals	Transmission rate of *Rfo* (%)	Number of both *Rfo* and *CRa* positive individuals
18CR1	1103	300	1	0.3	0
18CR2	2435	500	0	0	0
18CR3	6021	2000	5	0.25	3
18CR5	5026	2000	10	0.5	4

### Microspore culture

To obtain homozygous *CRa* individuals and accelerate the breeding process, microspore cultures were performed on ‘19CR1’, ‘19CR2’, ‘19CR3’, and ‘19CR4’ (Fig. S4, see online supplementary material). The results showed that the percentage of embryos in different combinations was quite different (Table 2). The ‘19CR1’ accounted for the highest percentage of embryos, followed by ‘19CR2’. Meanwhile, ‘19CR3’ was as low as 3% and no embryos were formed in ‘19CR4’. The difference in the percentage of embryos could be attributed to the differences in the genotype as both ‘19CR1’ and ‘19CR2’ coming from the male parent ‘18CR3’, while both ‘19CR3’ and ‘19CR4’ came from male parent ‘18CR5’. Finally, 47 and 35 viable lines were obtained from ‘19CR1’ and ‘19CR2’, respectively, among which 22 (named as ‘19CR1–1’–‘19CR1–22’) and 14 (named as ‘19CR2–1’–‘19CR2–14’) lines contained *CRa*, respectively. After cultured at differentiation medium, 3–6 seedlings were differentiated from each positive line, and finally, 89 and 64 positive seedlings of ‘19CR1’ and ‘19CR2’ were obtained, respectively. Additionally, 24 *CRa*-negative seedlings were also obtained, which were named as ‘19CK1’–‘19CK24’. All seedlings were treated with colchicine and then planted in the greenhouse for the next experiment.

**Table 2:** Microspore culture from four cross combinations containing *CRa*.

Cross combination	Number of buds	Number of embryos	Percentage of embryos (%)	Number of viable lines	Percentage of viable haploid lines	Number of *CRa* positive haploid lines	Percentage of *CRa* positive haploid lines (%)	Total number of *CRa* positive individuals
19CR1	75	164	219	47	0.28	22	47	89
19CR2	90	80	89	35	0.44	14	40	63
19CR3	60	2	3	0	0	0	0	0
19CR4	60	0	0	0	0	0	0	0

**Figure 1:**
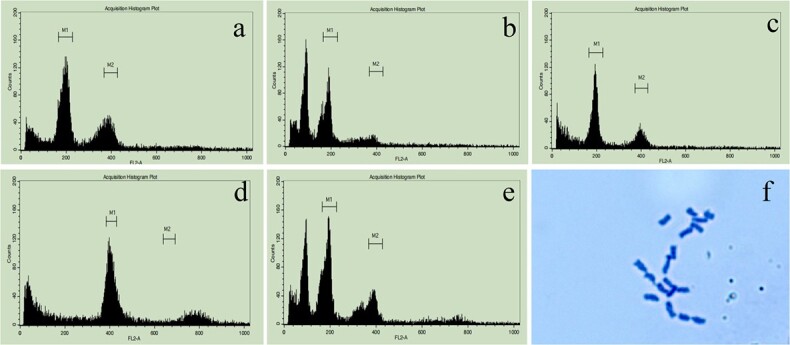
Flow cytometric determination of DNA content karyotype analysis of microspore individuals. **a** ‘ZM1’ (control). **b** Haploid. **c** Diploid. **d** Tetraploid. **e** Chimera. **f** ‘19CR2–7’.

### Cell ploidy, karyotype, and fertility identification of microspore individuals

The cell ploidy of all individuals was detected by flow cytometry (FCM) (Thermo, USA) after being treated with colchicine. The results showed that there were 58 diploids, 10 tetraploids and three chimeras in ‘19CR1’; and eight haploids, 28 diploids, six tetraploids and three chimeras in ‘19CR2’ (Table S1, see online supplementary material; Fig. 1a–e). Karyotype analysis result showed that ‘19CR2–7’ contained 18 chromosomes, indicating that this individual was diploid (Fig. 1f). In flowering plants, all haploids were sterile, and the other individuals among different ploidy could produce pollen normally (Fig. S5, see online supplementary material). After selfing, most individuals could bear seeds. However, the self-seed setting rate of individuals with different ploidy varied greatly, and the seed setting rate of diploids was much higher than that of tetraploids (Table 3). Not only that, but the seed setting rate of the combinations between diploids was also much higher than that of the combinations between diploid and tetraploid (Table 4).

**Table 3:** Seed setting statistics of self-bred microspore individuals of ‘19CR1’ with different ploidy.

Individual number	Number of seed pods	Number of seeds	Seed setting\rate (%)	Average seed setting rate (%)	Plant ploidy
19CR1–3-2	43	88	2.05	6.2	Diploid
19CR1–5-2	34	339	9.97		
19CR1–6-2	76	600	7.89		
19CR1–6-3	36	260	7.22		
19CR1–7-1	25	143	5.72		
19CR1–7-2	65	281	4.32		
19CR1–7-3	13	81	6.23		
19CR1–1-2	14	2	0.00	0.63	Tetraploid
19CR1–2-1	20	0	0.14		
19CR1–2-3	100	198	1.98		
19CR1–3-4	28	47	1.68		
19CR1–4-4	16	9	0.56		
19CR1–15-1	14	0	0.00		
19CR1–15–2	25	1	0.04		

**Table 4:** Seed setting statistics of different cross combinations between microspore individuals of ‘19CR1’.

Cross combination	Number of seed pods	Number of seeds	Seed setting rate (%)	Average seed setting rate (%)
19CR1–5-2 (Diploid) × 19CR1–5-3 (Diploid)	44	236	5.36	4.03
19CR1–7-1 (Diploid) × 19CR1–8-1 (Diploid)	18	100	5.56	
19CR1–9-2 (Diploid) × 19CR1–14-1 (Diploid)	57	66	1.16	
19CR1–9-3 (Diploid) × 19CR1–2-1 (Tetraploid)	73	1	0.01	0.29
19CR1–9-4 (Diploid) × 19CR1–2-1 (Tetraploid)	49	2	0.04	
19CR1–11–2 (Diploid) × 19CR1–15–2 (Tetraploid)	42	38	0.9	
19CR1–14-1 (Diploid) × 19CR1–15–2 (Tetraploid)	63	1	0.02	
19CR1–2-1 (Tetraploid) × 19CR1–14-1 (Diploid)	73	34	0.47	

### Flower’s morphology and pollen vigor identification of microspore individuals

The flower’s morphology among different ploidy was quite different. Compared with diploid and chimera plants, flowers from tetraploid plants had slightly wider petals and the color was much darker. In addition, the flower spread of diploid individuals was much less than that of tetraploid and chimera, while there were no significant differences between that of tetraploid and chimera plants (Figs. S6a–c and S7, see online supplementary material).

The pollen vigor were measured to study the effect of pollen vigor on the seed setting rate of microspore individuals. The results indicated that although the pollen vigor of diploids, tetraploids and chimeras was relatively high, there was no statistically significant differences among them (Fig. S6d–f, see online supplementary material). From this, we concluded that the difference in seed setting rate among different individuals was mainly affected by cell ploidy.

### Acquisition of distant hybridization offspring

To maximize the survival rate of F_1_ individuals, all the seed pods produced after the cross was used for embryo rescue (Fig. S8, see online supplementary material). Finally, 16 crossing progenies were obtained and were named as ‘16QOR1’ to ‘16QOR16’. The pod setting rate, embryo rate, and emergence rate of the four hybrid combinations were between 19.58–81.25%, 0.46–3.04%, and 1.20–8.57% (Table 5), respectively, which indicated that hybrid compatibility had significant differences among different hybrid combinations. Although the combination ‘16Q73’ × ‘BR1’ had the highest pod setting rate, it also had the lowest embryo rate and emergence rate among the four hybrid combinations. The embryo rate and the emergence rate of the ‘16Q235’ × ‘BR1’ were the highest, indicating this combination had the best hybridization compatibility. Therefore, we chose ‘16Q235’ as the recurrent parent for the backcrosses in the following experiments.

Based on the results of the identified molecular markers, cell ploidy and morphology, ‘16QOR4’ and ‘16QOR12’ were selected as donor parents to cross with ‘16Q235’. BC_1_ individuals were obtained by both embryo rescue and natural fruiting. The results showed that the embryo rate of BC_1_ obtained from embryo rescue (3.03%) was lower than the seed setting rate of BC_1_ obtained from natural fruiting (5.56%) and the emergence rate of BC_1_ obtained from embryo rescue (5.04%) was also lower than that of BC_1_ obtained from natural fruiting (17.5%) (Tables 6 and 7). In addition, we also found the emergence rate/seed setting rate of positive crossing (‘16Q235’ was used as female) was lower than that of negative crossing regardless of the method used. For convenience, all BC_1_ individuals obtained by embryo rescue were named as ‘ZE35–1’–‘ZE35–51’, while the individuals obtained by natural fruiting were named as ‘ZE36–1’–‘ZE36–14’. After the molecular marker was identified, all positive BC_1_ individuals were planted in the greenhouse for producing BC_2_ in the next year.

**Table 5:** Rescued embryo from different cross combinations between *Brassica oleracea* and *Brassicarapa*.

Cross combination	Number of pollinated flowers	Number of pods	Pod setting rate (%)	Number of embryos	Embryo rate (%)	Number of seedlings	Emergence rate (%)
16Q73 × BR1	224	182	81.25	83	0.46	1	1.20
16Q926 × BR1	133	39	29.32	28	0.72	1	3.57
16Q2038 × BR1	383	75	19.58	74	0.99	2	2.70
16Q235 × BR1	108	46	42.59	140	3.04	12	8.57
Total	848	342	40.33	325	0.95	16	4.68

**Table 6:** Creation of BC_1_ by embryo rescue.

Cross combination	Number of pods	Number of embryos	Embryo rate (%)	Number of seedlings	Emergence rate (%)
16Q235 × 16QOR4	154	267	1.73	6	2.25
16Q235 × 16QOR12	86	142	1.65	3	2.11
16QOR4 × 16Q235	55	367	6.67	22	5.99
16QOR12 × 16Q235	39	235	6.03	20	8.51
Total	334	1011	3.03	51	5.04

**Table 7:** Creation of BC_1_ by natural fruiting.

Cross combination	Number of pollinated flowers	Number of pods	Pod setting rate (%)	Seed number	Seed setting rate (%)	Number of seedlings	Emergence rate (%)
16Q235× 16QOR4	476	241	50.63	6	2.49	1	16.67
16Q235× 16QOR12	430	370	86.05	3	0.81	0	0
16QOR4× 16Q235	493	346	70.18	36	10.40	6	16.67
16QOR12× 16Q235	622	483	77.65	35	7.25	7	20
Total	2021	1440	71.25	80	5.56	14	17.50

Similar to BC_1_, BC_2_ individuals were also obtained by both embryo rescue and natural fruiting. Overall, the embryo or seed setting rate of BC_2_ from embryo rescue was higher than that from natural fruiting (Table 8). This fact was contrary to that of BC_1_. In addition, we noticed the emergence rate of ‘16Q235’ × ‘ZE35’ was lower than ‘ZE35’ × ‘16Q235’, which was the same as that of BC_1_. These results guided distant hybridization to a certain extent. For convenience, 26 BC_2_ individuals from ‘16Q235’ × ‘ZE35’ and ‘ZE35’ × ‘16Q235’, three individuals from ‘16Q235’ × ‘ZE36’, and 21 individuals from ‘16Q235’ × ‘ZE35’ obtained from natural fruiting were named as ‘ZF1’–‘ZF26’, ‘ZG1’–‘ZG3’, and ‘ZH1’–‘ZH21’, respectively.

**Table 8:** Creation of BC_2_ by embryo rescue and natural fruiting.

Cross combination	Number of pods	Number of embryos or seeds	Embryo rate or seed setting rate	Number of seedlings	Emergence rate (%)	Obtaining method
16Q235 × ZE35	420	105	0.25	7	6.67	Embryo rescue
ZE35 × 16Q235	446	442	0.99	19	4.30	Embryo rescue
16Q235 × ZE36	120	375	3.13	3	0.80	Embryo rescue
16Q235 × ZE35	256	28	0.11	21	75	Natural fruiting

### Molecular marker, ploidy, and karyotypes identification of distant hybridization offspring

**Figure 2:**
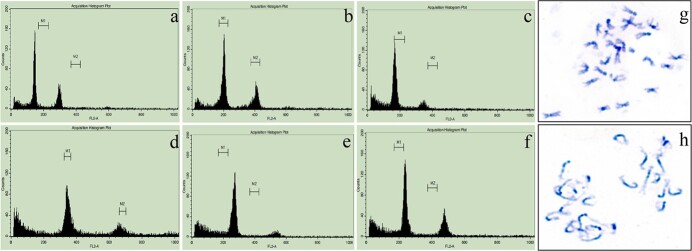
Flow cytometric determination of DNA content and karyotype analysis of distant hybridization offspring. **a** ‘BR1’. **b** ‘16Q235’. **c** Allodiploid F_1_. **d** Allotetraploid F_1_. **e** Triploid BC_1_. **f** BC_2_. **g** ‘ZF1’. **h** ‘ZF10’.

Molecular markers KBrH129J18, ZM91, and cnu_m090a, which were linked to *CRa*, *CRb*, and *Pb8.1*, respectively, were used to identify CR genes of F_1_, BC_1_, and BC_2_ individuals. The relative DNA content of the positive individuals of F_1_, BC_1_, and BC_2_ containing CR genes was detected by FCM, where ‘16Q235’ was used as the internal standard. For F_1_, all the individuals contained all of *CRa*, *CRb*, and *Pb8.1* (Fig. S9, see online supplementary material), and six individuals of them were identified as allotetraploid (Fig. 2d), whose chromosomal composition was speculated as AACC (Table S2, see online supplementary material). Other F_1_ individuals were identified as allodiploid (Fig. 2c), and the chromosomal composition of which was speculated as AC (Table S2, see online supplementary material). For BC_1_, 18 individuals obtained from embryo rescue contained all three CR genes (Fig. S10, see online supplementary material); however, only four of them were of theoretical chromosomal composition (Fig. 2e; Table S2, see online supplementary material). For BC_2_, 16 individuals contained at least one CR gene, and the chromosome composition of them conforms to the theoretical value of BC_2_ (Figs. S2f and S11; Table S2, see online supplementary material). The result of karyotype analysis showed that both ‘ZF1’ and ‘ZF10’ contained 25 chromosomes, which was consistent with the results of ploidy analysis (Fig. 2g and h). Among these positive individuals of BC_2_, seven of them contained three CR genes, five contained both *CRa* and *CRb*, and four contained only *Pb8.1*. Notably, all individuals containing *CRa* also contained *CRb*, indicating the linkage of these two genes, which was consistent with the previous report [17]. The positive rates of individuals from F_1_ to BC_2_ were also calculated. The 100% of the positive rate was achieved in F_1_ while only 7.84 and 7.14% of the positive rate were achieved by the rescue embryo and natural fruiting, respectively, in BC_1_. In BC_2_, 42.31% of the positive rate of individuals containing *CRa* and *CRb* was obtained from rescue embryo, which was the same as that of individuals containing *Pb8.1*. However, the positive rate of individuals containing *CRa* and *CRb* and obtained from natural fruiting was only 4.76%. From these results, we concluded that it is easier to break through the barriers of distant hybridization through embryo rescue.

### Phenotypical characteristics of distant hybridization offspring

**Figure 3:**
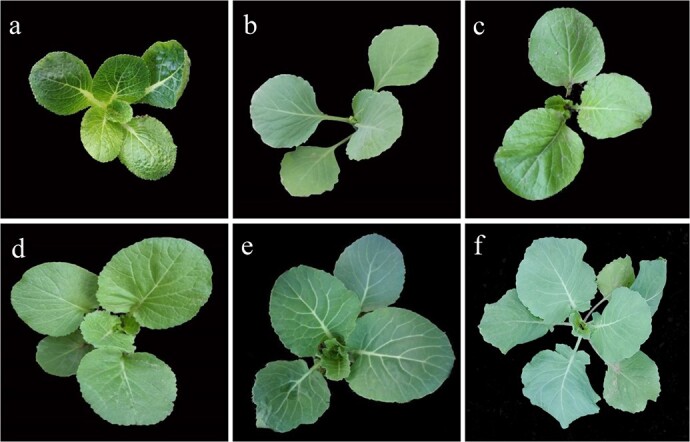
Morphological variations of seedling of allotetraploid plants (F_1_, BC_1_, BC_2_, and their parents). **a** ‘16Q235’. **b** ‘BR1’. **c** Allodiploid F_1_**d** Allotetraploid F_1_. **e** BC_1_. **f** BC_2._

To further understand the phenotypic differences amongst different genotypes, we observed the morphological characteristics of F_1_, BC_1_, and BC_2_ during their growth and development stage, and compared against their parents. As shown in Table 9 and Fig. 3, all the traits except for flower color in allotetraploid F_1_ were intermediate between the parents. It was almost indistinguishable between BC_1_ and ‘16Q235’ in leaf color, wax powder, leaf trichome, and pistil length while the flower width and stamen length in BC_1_ were further reverted to ‘16Q235’. However, their leaf shape, leaf margin, bud length, and flower color were still similar to allotetraploid F_1_. Compared with BC_1_, the overall phenotypical characters of BC_2_ were more similar to that of ‘16Q235’. Except for leaf shape, leaf margin, flower color, and stamen length, other traits were all restored to ‘16Q235’.

**Table 9:** Phenotypical characteristics of ‘16Q235’, ‘BR1’, allotetraploid F_1_, BC_1_, and BC_2_.

Phenotype	16Q235	BR1	Allotetraploid F_1_	BC_1_	BC_2_
Leaf shape	Sector	Oblong	Oval	Oval	Oval
Leaf color	Green	Light green	Chartreuse	Green	Green
Leaf margin	Entire	Serrated	Repand	Repand	Repand
Wax powder	Possession	Null	Little	Possession	Possession
Leaf trichome	Null	Possession	Little	Null	Null
Bud length	1.10 ± 0.15a	0.51 ± 0.02d	0.75 ± 0.01b	0.72 ± 0.35b	1.05 ± 0.23a
Flower color	Light yellow	Yellow	Yellow	Yellow	Yellow
Flower width	2.6 × 2.58	1.45 × 1.39	1.84 × 1.70	2.10 × 2.05	2.56 × 2.48
Stamen length	1.28 ± 0.34a	0.77 ± 0.11c	1.00 ± 0.07b	1.06 ± 0.22a	1.08 ± 0.14a
Pistil length	1.06 ± 0.13a	0.65 ± 0.01b	0.97 ± 0.06a	1.05 ± 0.24b	1.07 ± 0.11a

In the process of creating BC_1_, we found a very interesting phenomenon; one positive individual (ZE36–1) that came from natural fruiting was identified as a tetraploid. Because its female parent was ‘16Q235’, we speculate its chromosome composition was ACCC (Table S2, see online supplementary material). To further study the characteristics of this plant, we compared its traits with these of other BC_1_ individuals. As shown in Figs. S12 and S13 (see online supplementary material), the leaves of ‘ZE36–1’ were slightly deformed, while other characteristics including flower spread, flower color, pollen vigor, and spike shape were not significantly different from other BC_1_ individuals.

### Resistance and genotype

**Figure 4:**
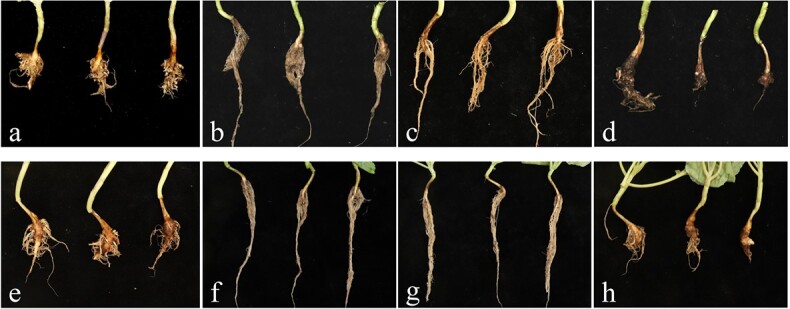
Resistance test to ‘ZM1’, *CRa*-positive and negative microspore plants, which inoculated with *P. brassicae* from Wulong. **a**, **e** ‘ZM1’. **b**, **f** ‘19CR1–10’. **c**, **g** ‘19CR2–7’. **d**, **h** ‘19CK2’. **a**, **b**, **c**, and **d** were inoculated with *P. brassicae* from Changyang. **e**, **f**, **g**, and **h** were inoculated with *P. brassicae* from Wulong.

**Figure 5:**
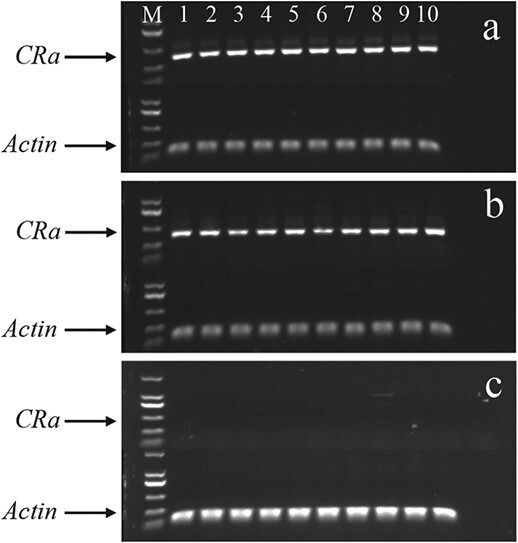
Semi-quantitative RT-PCR analysis of *CRa* for ‘19CR1–10’, ‘19CR2–7’, and ‘19CK2’. M, Marker. 1–10 individuals of corresponding lines. **a** ‘19CR1–10’. **b** ‘19CR2–7’. **c** ‘19CK2’

To explore the resistance of microspore plants, the self-progenies of ‘19CR1–10’, ‘19CR2–7’, and ‘19CK2’ were inoculated with *P. brassicae* collected from Changyang and Wulong, while ‘ZM1’ was used as a susceptible control. As shown in Table 10, the resistance of *CRa*-positive seedlings was significantly improved compared with *CRa*-negative seedlings (Fig. 4). Results of RT-PCR indicated the expression level of *CRa* in ‘19CR1–10’ and ‘19CR2–7’, which were inoculated with *P. brassicae* from Wulong, was very high while no expression of *CRa* was observed in ‘19CK2’ (Fig. 5).

**Table 10:** Resistance test of *CRa*-positive and -negative microspore seedlings.

Materials	Disease index	Resistance assessment^a^	Origin of *P. brassicae*
ZM1	100.00	HS	Changyang
19CR1–10	34.17	S	Changyang
19CR2–7	30.00	MR	Changyang
19CK2	91.67	HS	Changyang
ZM1	97.50	HS	Wulong
19CR1–10	7.14	R	Wulong
19CR2–7	29.76	MR	Wulong
19CK2	98.81	HS	Wulong

a HR, highly resistant; HS, highly susceptible; MR, moderate resistant; R, resistant; S, susceptible.

**Figure 6:**
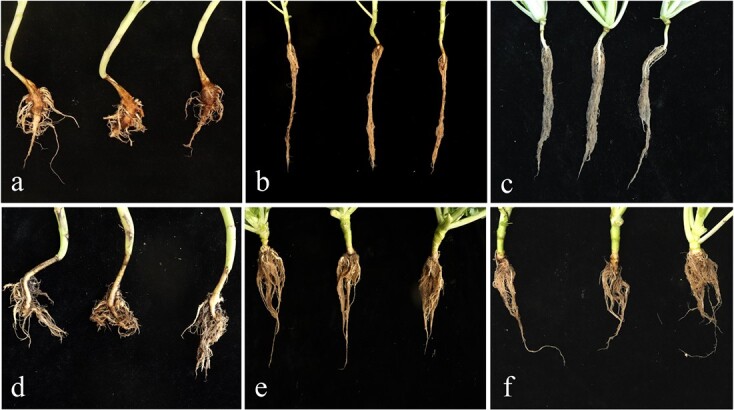
Identification of resistant genotypes against *P. brassicae*. **a** ‘ZM1’ (negative control). **b** ‘ZM2’ (positive control). **c** ‘BR1’. **d** ‘16Q235’. **e** BC_1_. **f** BC_2_.

Inoculation test was also performed in the progenies obtained from distant hybridization. Three individuals from both BC_1_ and BC_2_ which carried *CRa*, *CRb*, and *Pb8.1* and their parents were inoculated with *P. brassicae* collected from Changyang. ‘ZM1’ and ‘ZM2’ were used as susceptible and resistant control, respectively. The results revealed that both BC_1_ and BC_2_ showed high resistance to the disease (Table 11; Fig. 6), which suggested resistance strains with *CRa*, *CRb*, and *Pb8.1*. were successfully created.

**Table 11:** Resistant test of different genotypes.

Materials	Disease index	Resistance assessment^a^	Origin of *P. brassicae*
ZM1	97.50 ± 2.36	HS	Changyang
ZM2	7.14 ± 0.21	R	Changyang
BR1	4.49 ± 0.46	HR	Changyang
16Q235	58.33 ± 1.74	S	Changyang
BC_1_	3.57	HR	Changyang
BC_2_	4.49	HR	Changyang

a HR, highly resistant; HS, highly susceptible; MR, moderate resistant; R, resistant; S, susceptible.

### Identification of the *B. rapa*-derived fragments and determination of its insertion position in cabbage genome

**Figure 7:**
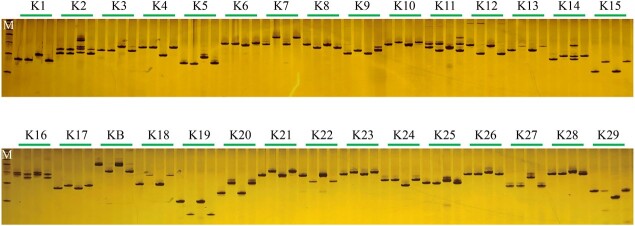
Identification of polymorphic DNA markers of exogenous fragment in cabbage genome. M, Marker. K1-F29, the name of InDel markers. KB, *CRa-*specific marker KBrH129J18. For each marker, the order of the samples was ‘19CR2–7’, ‘19CK2’, ‘BR1’, and cabbage.

**Figure 8:**
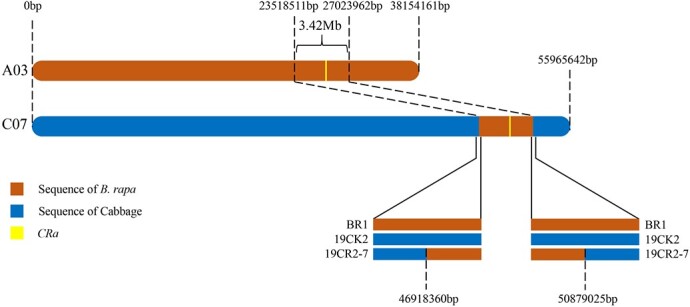
Model illustrating the introgression of an exogenous fragment from *Brassica rapa* into cabbage chromosome.

**Figure 9:**
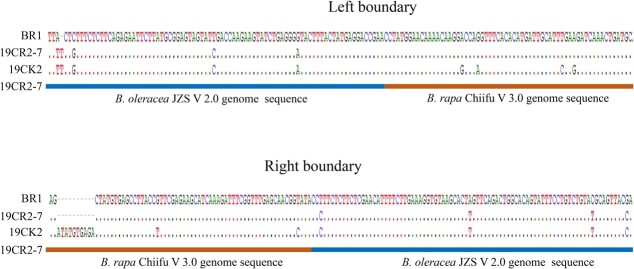
Left and right border sequences of the fragment from *Brassica rapa* in the cabbage genome.

**Figure 10:**
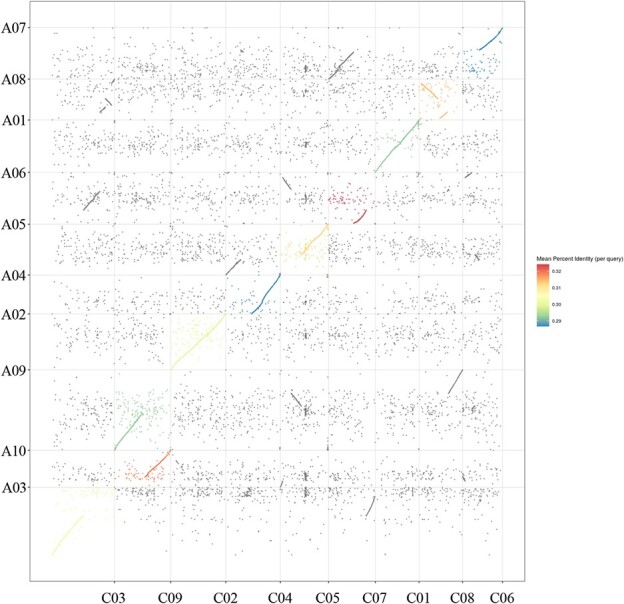
Segmental collinearity analysis of *Brassica rapa* and cabbage genomes.

**Figure 11:**
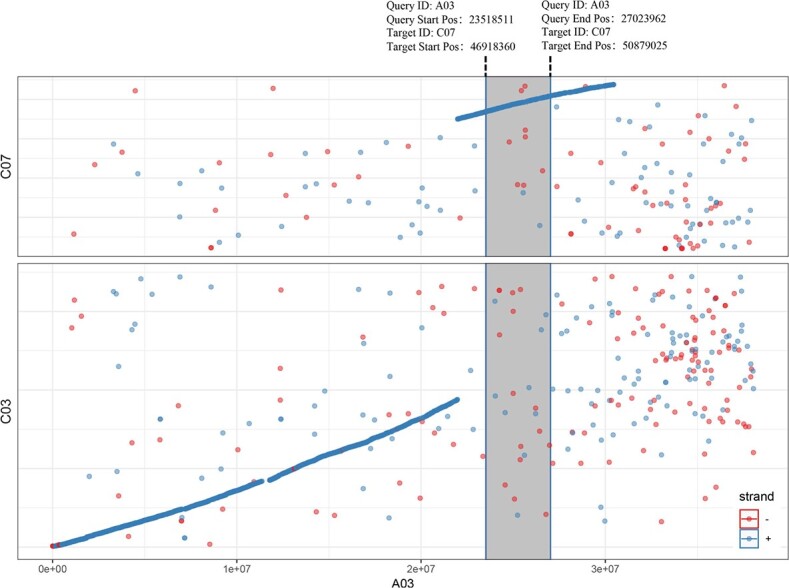
Analysis of collinearity between *Brassica rapa* A03 chromosome and corresponding chromosomes in cabbage genomes. The gray background indicates the position of the *B. rapa*-derived fragment inserted into the chromosome of cabbage.

**Figure 12:**
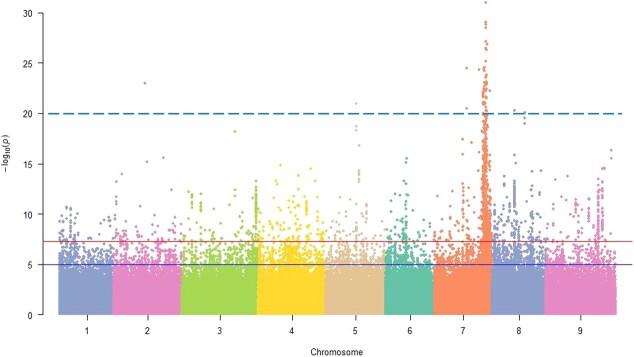
Manhattan plot showing genome-wide association study (GWAS) results for the location of *CRa*. The dotted line denotes the lower threshold for genome-wide significance.

To determine which part of the DNA fragment of the *B. rapa* genome was integrated into cabbage genome in the *CRa*-positive microspore individual, the genome of *CRa*-positive microspore individual was sequenced and the sequence data were compared with the sequences of both the *B. rapa* genome and the cabbage genome. Results showed that a fragment in the A03 chromosome from *B. rapa* was integrated into the cabbage genome (Table S3, see online supplementary material). To verify this and determine the location of the integration, 29 InDel markers in the range of 23–28 Mb of the A03 chromosome were developed (Table S4, see online supplementary material) and used to amplify the DNA samples from ‘19CR2–7’ (*CRa*-positive individual), ‘19CK2’ (*CRa*-negative individual), ‘BR1’ (*B. rapa*), and cabbage. As shown in Fig. 7, from K7 to K23, the amplification results of all primers have the same characteristics, that is, ‘19CR2–7’ and ‘BR1’ have the same band pattern, and ‘19CK2’ and cabbage also have the same band pattern. For other primers, the amplification results showed that ‘19CR2–7’, ‘19CK2’, and cabbage have the same band pattern, while ‘BR1’ has a special band pattern. Therefore, we speculate that the left boundary of the fragment was between K6 and K7, and the right boundary was between K23 and K24. Next, we designed a primer set LB1 (forward: 5’-GGAAACGAGAAAACGCAG-3′, reverse: 5’-ATGGAAGAAGGAATGAGC-3′) and RB1 (forward: 5’-TACTTCACATCCATCCAA-3′, reverse: 5’-CCGAGAACCAAAATAATC-3′) around the left and right boundary of the exogenous fragment to amplify ‘19CR2–7’, ‘19CK2’, and ‘BR1’. After sequencing the amplified products and comparing them with each other, we determined that the position of the fragment in the *B. rapa* genome was within the range of 23518511–27023962 bp in A03, while the corresponding position in the cabbage genome was within the range of 46918360–50879025 bp in C07 (Figs. 8 and 9). Interestingly, the result of the collinearity analysis between *B. rapa* and cabbage genomes showed that the region of 21983834–32826292 bp in A03 of *B. rapa* genome was homologous to the region of 45028469–55415234 bp in C07 of cabbage genome (Figs. 10 and 11). At the same time, the genome-wide association study (GWAS) was also performed to further determine the location of the *CRa* gene in the cabbage genome. As shown in Fig. 12, there was a significant association locus at the end of C07 in cabbage. The interval where the significance threshold was >20 was within the range of 49969353–49986020 bp (Table S5, see online supplementary material). These results suggested that the way of integration of exogenous fragment into the cabbage genome might be through HE.

For BC_2_ individuals obtained by distant hybridization, the same strategy was used to identify fragments from the *B. rapa* genome (Table S6, see online supplementary material), and the result showed that except A05 and A10, almost all the sequences in other chromosomes entered BC_2_. Subsequently, InDel markers were designed near the boundaries of fragments from A03, A05, and A09 to amplify DNA samples of ‘BR1’, ‘ZF8’, and ‘16Q235’ (Table S7, see online supplementary material). From Fig. S14 (see online supplementary material) we can speculate that all sequences in A03 and A09 were integrated into BC_2_, while a part of sequences in that of A05.

## Discussion

### Three CR genes from *B. rapa* were introduced into cabbage

Distant hybridization technology has been widely used for the introduction of excellent genes from close wild species or other species into target crops inbreeding. The rich germplasm resources of *Brassica* in the family Cruciferae provide material support for the achievement of distant hybridization. Bannerot *et al.* [52] transferred the cytoplasmic sterility traits from radish into the cabbage by distant hybridization. Mei *et al.* [53] used wild cabbage as a resistant donor to conduct distant hybridization with susceptible rapeseed and successfully created rape materials with resistance to sclerotinia. In addition, distant hybridization is also widely used in the cultivation of cruciferous CR varieties. Hagimori *et al.* [54] carried out somatic hybridization between broccoli and radish and obtained hybrid offspring with 36 chromosomes, which could be used as bridge germplasm for subsequent CR broccoli cultivation. Hasan and Rahman [55] obtained the *B. juncea* which was resistant to race 3 of *P. brassicae* using the method of distant hybridization. Liu *et al.* [56] identified three *B. rapa* with high resistance to Pb4 from 50 materials, which were then used as donor parents to cross with rapeseed, and obtained CR progenies. In this study, the *B. rapa* ‘BR1’ with resistance to race Pb4 was used as the male parent to conduct distant hybridization with ‘16Q235’ and finally, BC_2_ carrying three CR genes was created, which can be used for further CR breeding.

Hybridization between different species is complicated and often leads to failures at many stages starting from pollination incompatibility due to pre/post-fertilization barriers. Seed setting in a natural state may be prevented due to abortion of embryos during the early stage of development [57], although fertilization may take place. In our research, embryo rescue was proven to be a more efficient method to obtain positive individuals in the progress from F_1_ to BC_2_. Similar findings have also been reported by earlier researchers [58]. However, the survival rate of embryos was affected by parental genotypes [59]. Through comparing the different cross combinations in this study, the highest seeding rate of crosses between ‘16Q235’ and ‘BR1’ was found, which indicated that the compatibility was high between them. Therefore, to obtain backcross progenies more easily, we selected ‘16Q235’ as the recurrent parent for backcross.

At present, various CR genes have been mapped in *B. rapa* and many molecular markers linked to CR genes have been developed, which provides great convenience for the introduction of CR genes from *B. rapa* into the cabbage by distant hybridization. In this study, we successfully created the BC_2_ materials containing three CR genes, which can be used for the CR breeding of cabbage in the future. Our research provided a practical way for introducing other CR genes such as *Crr1* and *CRd*, etc. into cabbage genome through distant hybridization or other methods.

One of the characteristics of *P. brassicae* is the differentiation of physiological races, which is accompanied by a variation of virulence. The resistance of the cultivars with a single resistance gene is limited and may be overcome by *P. brassicae* over time. Therefore, pyramiding two or more resistance genes into one material to obtain broad-spectrum resistance is the main goal of CR breeding. Matsumoto *et al.* [60] have pyramided *CRa*, *CRc*, and *CRk* together by using hybridization and MAS, which improved the resistance of Chinese cabbage to clubroot. In this study, we introduced three CR genes from *B. rapa* into cabbage at the same time, which greatly improved the CR of cabbage, and provided the fundamental materials for CR breeding in the future.

### Microspore culture speeds up the CR breeding process

Cabbage is a cross-pollinated crop with an obvious hybridization advantage. Therefore, hybrid breeding has become the main breeding method in cabbage breeding and almost all cabbage varieties on the market are hybrids, which are usually produced by high-generation inbred lines. However, it is a time-consuming and labor-intensive process to obtain a high-generation inbred line by the traditional breeding method [61]. Because of this, microspore culture, a rapid breeding strategy has been developed and applied in various plant breeding. Lichter [62] successfully obtained embryoids and plants of *B. napus* by using microspore culture, which laid the foundation for the application of free microspore culture on *Brassica* crops. Since then, the technology became a very important method in *Brassica* breeding to produce DH lines, construct genetic maps, locate genes with important economic or agronomic value, develop markers for MAS and accelerate crop breeding progress [63, 64, 65]. In this study, we successfully obtained cabbage DH lines containing the homozygous CR gene in only 3 years, which greatly improved the efficiency of CR breeding in cabbage and laid a foundation for the commercial application of CR cabbage materials.

Microspore embryogenesis is usually affected by various factors including genotypes of donor plant, media composition, pre-treatments with different temperatures, developmental stage of the buds, culture incubation conditions and microspore density in culture; amongst which genotype is considered a predominant factor [66]. Under the same culture conditions, the embryo rate of ‘19CR3’ and ‘19CR4’ were much lower than those of ‘19CR1’ and ‘19CR2’, indicating the great influence of genotypes. To increase the embryo rate from different materials, it is necessary to optimize different factors for a particular genotype [67].

Spontaneous chromosome doubling of microspore plants is a common phenomenon in *Brassicas*. Gu *et al.* [68] reported that a large percentage of *B. oleracea* microspore-derived seedlings were diplodized spontaneously in the culture medium. Bhatia *et al.* [66] observed that more than 50% of the microspore plants were spontaneous diploids. In our study, after being treated with colchicine, 14% and 13% microspore individuals of ‘19CR1’ and ‘19CR2’ became tetraploids, respectively, which means that these individuals were diplodized spontaneously before being treated with colchicine. This phenomenon may provide a practical reference for future research on cabbage microspore breeding, and the high spontaneous doubling rate of microspore plants can also save time and workload for breeders.

Bhatia *et al.* [67] conducted microspore culture on cauliflower and obtained diploid and tetraploid plants. The survey results of agronomic and floral traits showed that the tetraploid lines had normal fertility and more than 50% economic yield as compared with the diploids. Furthermore, triploid hybrids were successfully produced by using tetraploid lines as the pollen parent and diploid CMS line as female. It was found that these hybrids have very good vigor with excellent curd characters. Thus, a breeding strategy based on polyploidy could be a convenient breeding program in cauliflower. In our research, we also obtained diploid and tetraploid plants. However, we found that although the pollen vigor of tetraploid plants was almost the same as that of diploid plants, the seed setting rate of tetraploid plants was very low when participating in selfing or crossing with diploid plants. Therefore, we thought that the polyploidy breeding strategy may not be implemented in cabbage at least in the short term. Chromosomal stability of tetraploid plants may be one of the important factors that affect seed setting rate. Whether the tetraploid chromosomes can be stabilized through continuous self-crossing for years and the seed setting rate could be improved needs to be further studied.

### HE is the theoretical basis for the success of distant hybridization

*CRa* is the first CR gene cloned in *B. rapa* ‘T136–8’, and it is located at about 25.37–25.75 Mb of the A03 chromosome [16]. This gene has been widely used in cruciferous crops up to now but has not been found in ordinary cabbage inbred lines. Therefore, we speculate that the *CRa* gene in the cabbage cultivars ‘17CR1’, ‘17CR2’, ‘17CR3’, and ‘17CR5’ should be introduced from the *B. rapa* material by the method of distant hybridization. As expected, we validated HE between *B. rapa* and cabbage by whole-genome resequencing of *CRa*-positive microspore individual and BC_2_ plant obtained from distant hybridization along with the molecular identification markers specific to the rearranged region. Karyotype analysis result showed that the cabbage DH line ‘19CR2–7’ contained 18 chromosomes, indicating that the exogenous fragment from *B. rapa* has been fully integrated into the cabbage genome and can be stably inherited. The finding revealed the possible mechanism of the introgression of the exogenous fragments into the target genome in the process of distant hybridization, which may provide an important direction for the application of exogenous fragments in breeding. Besides the fragment from A03, we also identified a few fragments that seems from *B. rapa* in the genome of ‘19CR2–7’ and the negative individual that does not contain *CRa* (Table S3, see online supplementary material). However, these fragments were short and discrete. Due to the high similarity between the cabbage genome and the *B. rapa* genome, some fragments in the cabbage genome might be identified as coming from the *B. rapa* genome. In this case, the short and discrete fragments should be the sequence of the cabbage itself. Therefore, the introgression of A03 into C07 should be the only homoeologous exchange between the parents and the corresponding fragment in C07 should have been lost during the creation of the DH lines.

In addition, we found that using Spades and Mummer to assemble and analyse resequencing data, respectively, can efficiently locate *B. rapa-*derived fragments that have been introduced into cabbage, which is a convenient method for data analysis of interest in related research fields.

Unlike *B. rapa*, clubroot resistance in cabbage is usually quantitative character controlled by multiple genes, and the rate of phenotypic variation explained by CR loci that have been mapped was relatively low. However, previous research indicated that the CR of cabbage materials ‘Tekila’ and ‘Kilaherb’, which both developed by Syngenta, was controlled by a major dominant resistance gene *Rcr7* [11]. The researchers further pointed out that the *Rcr7* possibly originates from a gene in chromosome A03 of *B. rapa*, according to a Syngenta patent (http://www.google.com/patents/EP1525317A1?cl=en) on ‘Clubroot Resistant *B. oleracea* Plants’. In view of this, we speculate that all cabbage varieties (‘17CR1’, ‘17CR2’, ‘17CR3’, and ‘17CR5’) containing *CRa* used in our study may be from the same source.

## Materials and methods

### Plant materials and artificial pollination

Five Ogura CMS CR cabbage varieties – ‘17CR1’, ‘17CR2’, ‘17CR3’, ‘17CR4’ and ‘17CR5’ – were collected from market. The *B. rapa* variety ‘BR1’, which carried *CRa*, *CRb*, and *Pb8.1*, and was resistant to race 4 of *P. brassicae* (Pb4), was purchased from the Nongbowang Seed Co., Ltd., Fuzhou, China. The Ogura CMS restorer ‘17Q8–514’ was provided by a previous researcher [32]. The cabbage inbred lines ‘ZM1’, ‘ZM2’, ‘18Q379’, and ‘18Q887’ were provided by the Institute of Vegetables and Flowers, Chinese Academy of Agricultural Sciences (IVF-CAAS), among which, ‘ZM1’ and ‘ZM2’ were used as susceptible and resistant control in the inoculation experiment, respectively.

**Figure 13:**
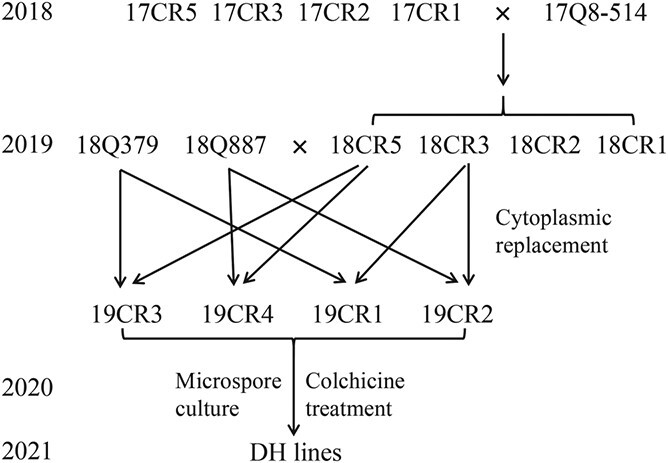
The pedigree map illustrating the creation process of cabbage DH lines.

In the spring of 2018, the cross combinations of ‘17CR1’ × ‘17Q8–514’, ‘17CR2’ × ‘17Q8–514’, ‘17CR3’ × ‘17Q8–514’ and ‘17CR5’ × ‘17Q8–514’ were created by using ‘17Q8–514’ as a male, and the progenies of the cross combinations were named as ‘18CR1’, ‘18CR2’, ‘18CR3’ and ‘18CR5’, respectively. In the spring of 2019, the individuals of ‘18CR3’ and ‘18CR5’ that carried *CRa* and *Rfo* were used as males to cross with both ‘18Q379’ and ‘18Q887’. And for the convenience of description, the progenies of cross combinations ‘18Q379’ × ‘18CR3’, ‘18Q887’ × ‘18CR3’, ‘18Q379’ × ‘18CR5’ and ‘18Q887’ × ‘18CR5’ were named as ‘19CR1’, ‘19CR2’, ‘19CR3’ and ‘19CR4’, respectively (Fig. 13). For each combination, three individuals containing *CRa* and having relatively fine genetic backgrounds were selected and planted in the greenhouse for microspore culture next year. In the spring of 2021, all individuals of ‘19CR1’ and ‘19CR2’ obtained from microspore culture were selfed. In addition, different cross combinations were conducted among different individuals obtained from microspore culture based on their cell ploidy. When the seeds were mature, the number of selfed seeds of partial individuals and the number of seeds of all combinations were counted, and the seed setting rate was calculated.

To introduce CR genes from *B. rapa* into the cabbage genome, four distant hybridization combinations of ‘16Q73’ × ‘BR1’, ‘16Q926’ × ‘BR1’, ‘16Q2038’ × ‘BR1’ and ‘16Q235’ × ‘BR1’ were performed using artificial pollination. Allotetraploid positive F_1_ individuals identified by molecular markers and FCM were colonized in the greenhouse, which were used for backcrossing with ‘16Q235’ continuously to get BC_1_ and BC_2_ (Fig. S15, see online supplementary material). The artificial pollination in the bud stage was carried out in clear weather and the fresh pollen from the male parent was applied to the stigma of the female parent with removed stamens.

### DNA extraction and molecular marker identification

Plant genomic DNA was extracted from young leaves by minor modifications of the cetyltrimethylammonium bromide (CTAB) method [69]. DNA concentration was measured by Nanodrop 2000 and was diluted to working concentration (30–50 ng/μL). The *Rfo*-specific marker Rfo-11 (about 2.88 kb close with *Rfo*) was used to detect *Rfo* [32]; two *CRa*-specific markers, SC2930 (about 1.56 Mb close with *CRa*) and KBrH129J18 (about 1.54 Mb close with *CRa*), were used to detect *CRa* [15, 16, 70]; *CRb*-specific marker ZM91 (forward primer: 5’-TGCGCAAGAAAGTATTGTCG-3′, reverse primer: 5’-CGGAACCATAATGGCAAATC-3′) (included in *CRb*) and *Pb8.1*-specific marker cnu_m090a (forward primer: 5’-GCAAAGATCGGCGAAGAAGA-3′, reverse primer: 5’-TGCAGACACATTCGAACAAACA-3′) (about 1.4 cM close with *Pb8.1*) were used to detect *CRb* and *Pb8.1* by polymerase chain reaction (PCR). PCR reaction (10 μL) contained 1 μL of template DNA, 0.5 μL of both forward and reverse primers, 5 μL of PCR master mix (Vazyme, Nanjing, China), and 3 μL of ddH_2_O. PCR amplification was performed using Bio-Rad thermocycler (USA) using 5 min 94°C taq activation followed by 35 cycles of 30 s 94°C, 30 s 56°C, 45 s 72°C and a final 5 min 72°C extension. PCR amplicons of SC2930 and Rfo-11 were separated on 1% agarose gels and visualized under ultraviolet light, while PCR amplicons of KBrH129J18, ZM91 and cnu_m090a were separated by 8% polyacrylamide gel electrophoresis at 150 V for 70 min, followed by silver staining.

### Microspore culture to create cabbage DH lines

The cabbage flower buds with a length of 3–3.5 mm were collected at 9 a.m. and placed in a 100 mL beaker. Bud surface was sterilized by incubation in 75% ethanol for 30 s, 8% sodium hypochlorite for 9 min, followed by 3 min wash with sterile dH_2_O for three times. The buds were then transferred to a 10 mL test tube with a round bottom containing a small amount of B5 medium (3.21 g B5 and 130 g sucrose in 1 L dH_2_O, pH 5.9) and grounded with a glass rod. The free microspores were filtered into a 10 mL centrifuge tube through a nylon mesh with 45 μm aperture and centrifuged at 1000 rpm for 5 min. The supernatant was discarded and the microspore pellet was re-suspended in B5 medium for three times. The microspore was then suspended in NLN medium (1.77 g NLN and 130 g sucrose in 1 L dH_2_O, pH 5.9) and the concentration was measured using a hemocytometer and adjusted to 1 × 10^5^ cfu/mL. Subsequently, a 2-mL microspore suspension was added to each 60 mm × 15 mm culture glass dish containing a drop of activated carbon solution (0.5 g/L). The culture glass dishes were sealed with parafilm and initially incubated at 32.5°C in the dark for 24 h and then the temperature was reduced to 25°C for the remainder of the incubation. After 2–3 weeks, embryos developing from the microspores were successively transferred to grow for 3 weeks on solid B5 medium (3.21 g B5, 20 g sucrose, and 10 g agar in 1 L dH_2_O, pH 5.9), for 3 weeks on MS solid medium (4.43 g MS, 28 g sucrose, and 8 g agar in 1 L dH_2_O, pH 5.9), for 3 weeks on differentiation medium (4.43 g MS, 1 mg 6-BA, 0.1 mg NAA, 28 g sucrose, and 8 g agar in 1 L dH_2_O, pH 5.9), for 1 month on growth medium (4.43 g MS, 0.2 mg 6-BA, 0.1 mg NAA, 28 g sucrose, and 8 g agar in 1 L dH_2_O, pH 5.9) and for 2 weeks on rooting medium (4.43 g MS, 0.1 mg IBA, 0.1 mg NAA, 28 g sucrose, and 8 g agar in 1 L dH_2_O, pH 5.9). In the autumn 2020, the roots of the tissue-cultured seedlings were cut into 1–2 cm pieces and soaked in 0.4% colchicine solution for 3 h before planting in greenhouse.

### Embryo rescue

Embryo rescue was applied in the progress of distant hybridization. After pollination 15–20 days, the ovary was collected and disinfected using the following steps: 1 min soaked in 75% alcohol, 15 min in 8% sodium hypochlorite, and rinsed with sterile dH_2_O for three times. The ovary was then placed on sterile filter paper and was peeled with a scalpel. The ovules were removed and placed on a solid B5 medium, and incubated in a growth chamber at 25°C with 10 h light/day with light intensity at 2000 lx for 20–30 days. The surviving ovules were transferred to a growth medium and cultured until the seedlings grew. After subculture, the seedlings were transferred to a rooting medium to induce rooting. Finally, all seedlings were planted in the greenhouse.

To double the chromosomes of the F_1_ seedlings, the F_1_ roots were also treated with colchicine solution and cultured with the same protocol used for microspore culture.

### Ploidy identifcation and cytological analysis

The relative DNA content of F_1_, BC_1_, and BC_2_ individuals obtained in the progress of distant hybridization as well as all positive individuals containing *CRa* developed from microspore culture were measured by FCM. The ploidy of the plant was determined based on the FCM results. The FCM test procedure followed the method modified by Doležel [71]. Briefly, the leaves from the young plant was collected and placed in a glass culture petri dish containing 2 mL cell lysate and shredded in one direction with a blade for 5 min. The tissue residues were removed using a 38 μm filter and the filtrate was centrifuged at 500 rpm for 4 min. The supernatant was discarded and the pellet was suspended in 500 μL propidium iodide (PI) solution (50 ug/mL) and incubated on ice for 30 min. The relative DNA content of each sample was measured by FCM and the ‘16Q235’ was used as the control sample. Each sample was tested with 10 000–20 000 cell particles.

For meiotic analysis, root tips of ‘19CR2–7’, ‘ZF1’, and ‘ZF10’ were collected and treated with 8-hydroxyquinoline for 3–5 h, 0.075 M/L KCl solution for 30 min, 2.5% enzyme mix for 1–2 h at room temperature. Then, the samples were rinsed with distilled water for 2–3 times and stained with Giemsa solution. Chromosome pairing at diakinesis and chromosome segregation at anaphase were observed and the number of chromosomes were counted.

### Plant morphology observation and data analysis

In the whole period of distant hybridization, the morphological characters and related data including leaf shape, leaf color, leaf margin, leaf epidermal waxiness, leaf epidermal villi, flower buds and floral organ traits of ‘BR1’, ‘16Q235’, F_1_, BC_1_, and BC_2_ were observed and recorded. The flowers spread for individuals of ‘19CR1’ and ‘19CR2’ obtained from microspore culture with different ploidy were statistically analysed. Nine individuals from diploids or tetraploids, and four individuals from chimera were selected for statistical analysis, nine flowers from each individual were measured. Subsequently, the pollen vigor of microspore individuals of ‘19CR1’ with different cell ploidy was assessed. Briefly, freshly opened flowers were collected at 9 a.m. and the pollen grains were released on a microscope slide and stained by alexander staining solution and analysed using microscopy.

### Inoculation test for plants cultured from microspore and distant hybridization

The *P. brassicae* used for artificial inoculation identification in this study was collected from Changyang, Hubei Province, and Wulong, Chongqing Municipality, China, which all identified as race 4 based on the differential classification of the Williams system [72]. The resting spore inoculum was prepared according to a previous study [73] with some modifications. Clubs in distilled water were homogenized with a blender. The slurry was filtered with eight layers of cheesecloth, and the suspension was centrifuged at 600 rpm for 10 min. The supernatant was transferred to a new tube and centrifuged at 3500 rpm for 10 min. The resulting sediment was washed three times with sterile water and finally, the concentration of resting spore in suspension was measured by a hemocytometer, and adjusted to 2 × 10^7^ cfu/mL. For each seedling, 2 mL resting spore suspension was injected into the bottom of the stem in the soil. Disease severity of roots was evaluated 42 days after inoculation (DAI) on a scale of 1 to 4 based on the following standard [74]: 0, normal root growth; 1, some small galls on the lateral roots; 2, slight clubs on the taproot or medium clubs on the lateral roots; 3, large clubs on the taproot; 4, severe galls on the taproot with almost no lateral roots. The disease index (DI) was calculated as $DI=\frac{\Sigma (n\times a)}{N}\times 4\times 100$, where *n* represents the number of individuals from each grade, *a* represents the corresponding disease grades (0–4), *N* represents the total number of individuals. The resistance was evaluated with the following standard: DI = 0, immune (I); 0 < DI ≤ 5, highly resistant (HR); 5 < DI ≤ 20, resistant (R); 20 < DI ≤ 30, moderately resistant (MR); 30 < DI ≤ 60, susceptible (S); and DI > 60, highly susceptible (HS).

### Semi-quantitative RT-PCR

After disease severity assessment, total RNA from the roots was extracted by using R318–50-Polysaccharide/polyphenol Plant Total RNA Mini Kit following the supplier’s instructions (GeneBetter Biotech, Beijing, China). The cDNA was reverse transcribed with the HiScript® III 1st Strand cDNA Synthesis Kit (Vazyme, Nanjing, China). The expression of *CRa* was quantified by RT-PCR using primer set ‘ZM78’ (forward primer: 5’-TGAACTCATACAGGGGAT-3′, reverse primer: 5’-CCAGCTTGTTTAAAACAC-3′), which were designed based the sequence of the cloned *CRa*. The expression of β-*actin* (forward primer: 5’-CCAGAGGTCTTGTTCCAGCCATC-3′, reverse primer: 5’-GTTCCACCACTGAGCACAATGTTAC-3′) was used as the internal control.

### Resequencing of individuals obtained by microspore and distant hybridization

To determine the position of *CRa* in the cabbage genome and explore the principle of the recombination of foreign fragments, a *CRa*-positive microspore individual (19CR2–7), a *CRa*-negative microspore individual (19CK2) and a BC_2_ individual (ZF8) obtained by distant hybridization were selected for sequencing. The sequenced data were assembled with Spades (http://cab.spbu.ru/software/spades/) and then compared with the *B. rapa* genome by using Mummer (http://mummer.sourceforge.net/). Additionally, collinearity analysis was performed for whole genomes of cabbage and *B. rapa* (http://brassicadb.cn/#/Download/) by using Mummer.

### A GWAS to identify the location of *CRa* in cabbage genome

There were 196 cabbage accessions used for GWAS, five of which contained *CRa* and the rest do not contain *CRa*. Among these accessions, the resequencing data of 85 and 96 accessions were obtained from NCBI Sequence Read Archive PRJNA700684 and SRP071086, respectively, while the resequencing data of other accessions were obtained by resequencing (Table S8, see online supplementary material)*.* The resequencing data were mapped to the *B. oleracea* (http://bogdb.com/genome/round_cabbage) genomes by using the BWA program. The detection of raw SNPs between the reference genome and sequenced samples was performed by using SAMtools software, through which the bam format file was drawn. Based on the bam file, the HaplotypeCaller model of the GATK software was used to perform SNP detection on a single sample to generate the original VCF file; the CombineVariants function of the GATK software was used to merge the VCF files of a single sample, and then the Genotype VCFs function was used for population SNP detection. The Variant Filtration function of the GATK software was used for population SNP filtering. Minor allele frequency (MAF) and missing data percentage (%) each were >5% and <30% in SNP matrix filtering result. GWAS was performed using the GEMMA program and the Mixed Linear Model was used to detect the significance of association between traits and genetic markers. The results of the association analysis were displayed in a Manhattan chart, in which the significance threshold was set to 20.

## Acknowledgements

The work was supported by the National Natural Science Foundation of China (Grant No. 31801876), Science and Technology Innovation Program of the Chinese Academy of Agricultural Sciences (CAAS-ASTIP-IVFCAAS), China Agriculture Research System of MOF and MARA (CARS−23), the Central Public-interest Scientific Institution Basal Research Fund (Y2021XK18), and Major Science and Technology Projects of Inner Mongolia Autonomous Region (2021ZD0001).

## Author contributions

Mi.Z. designed and performed the experiments, analysed the data, and drafted the manuscript; L.Y., Y.Z., and Mu.Z. collected data; H.L., J.J., and X.H. contributed to data analysis, figure plotting and table making; Z.L., F.H., Z.F., and Y.W. carefully revised the manuscript. All authors approved the final manuscript.

## Data availability

All data supporting our findings are available in the manuscript file or from the corresponding author upon request.

## Conflict of interest

The authors declare no competing interests.

## Supplementary data

Supplementary data is available at *Horticulture Research* online.

## Supplementary Material

Web_Material_uhac195
