# Supplementary figures and images for: Introgression of clubroot resistant gene into Brassica oleracea L. from Brassica rapa based on homoeologous exchange
Source: Hortic Res. 2022 Aug 30;9:uhac195. doi: 10.1093/hr/uhac195 (PMC10167419; doi:10.1093/hr/uhac195)

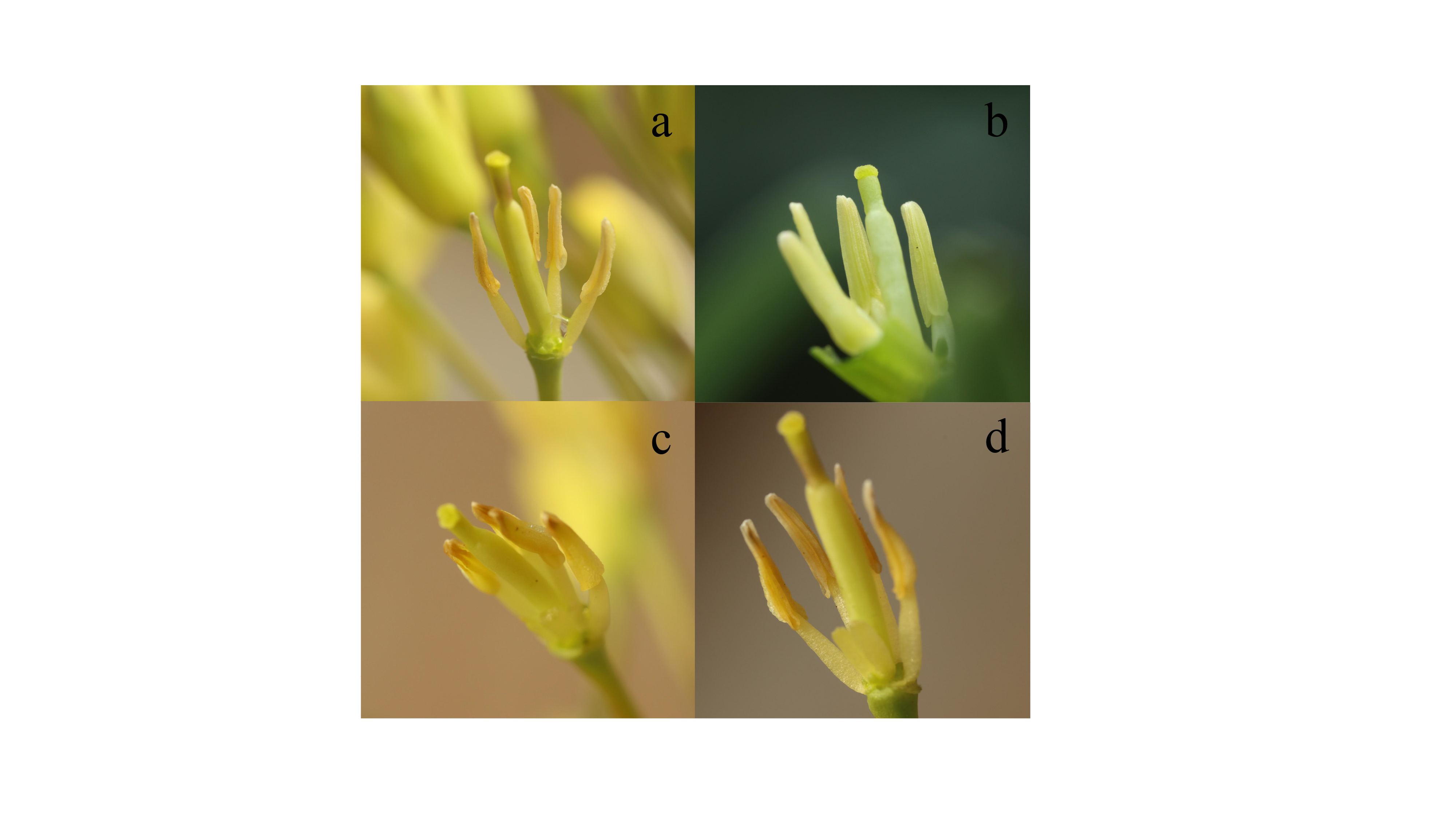

Supplement: Web_Material_uhac195 [file web_material_uhac195.zip › Fig. S1.jpg]

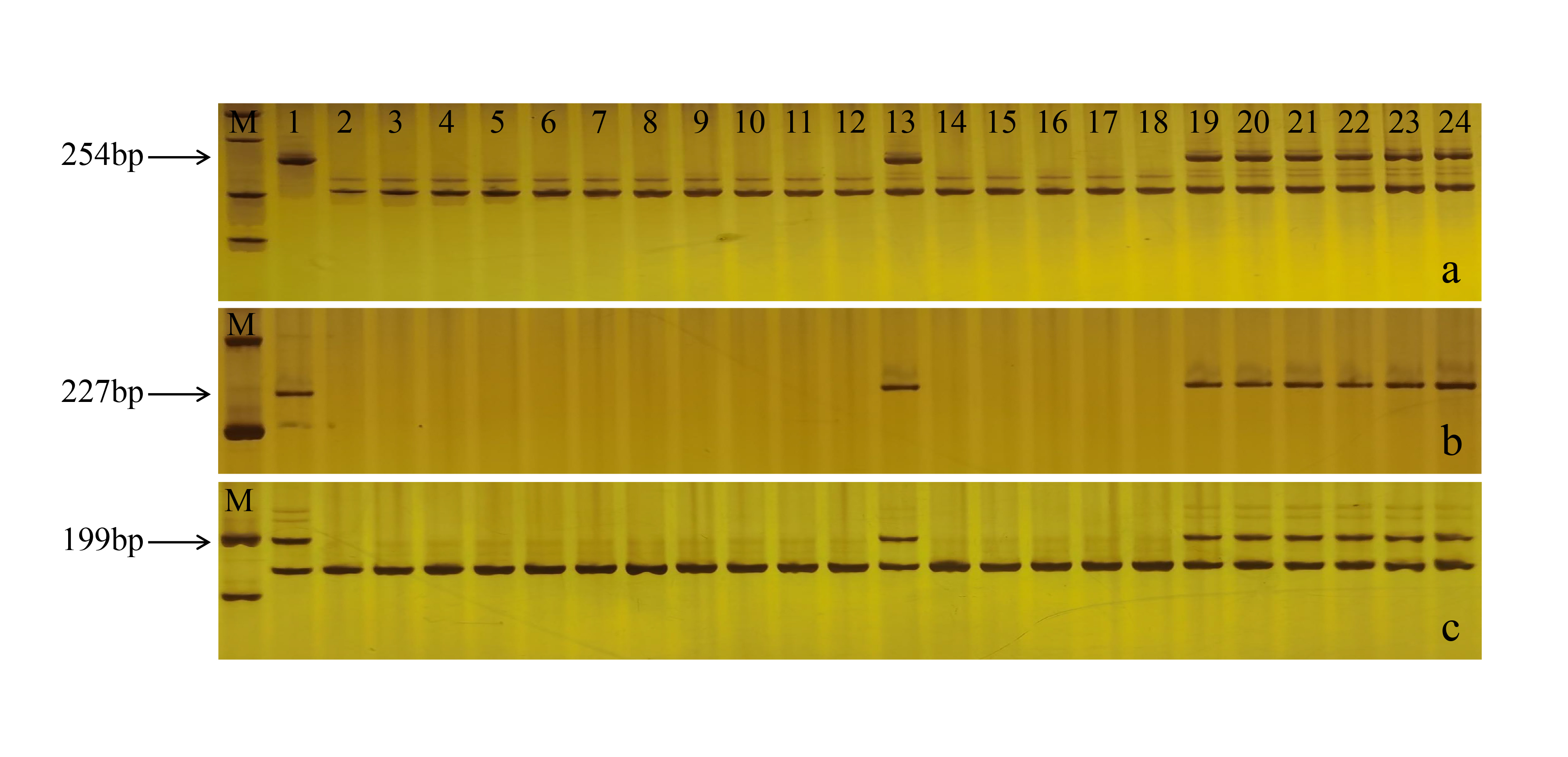

Supplement: Web_Material_uhac195 [file web_material_uhac195.zip › Fig. S10.jpg]

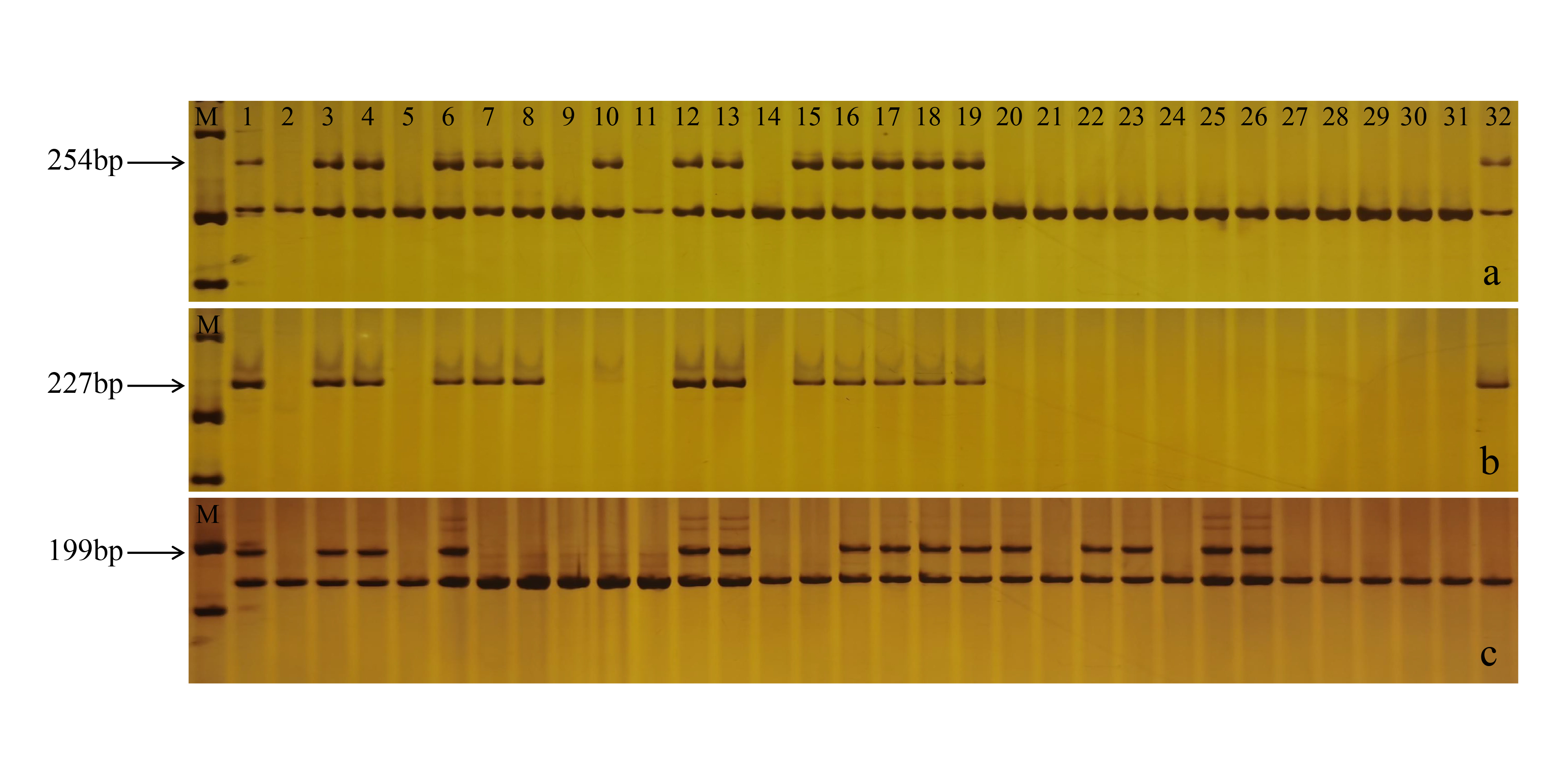

Supplement: Web_Material_uhac195 [file web_material_uhac195.zip › Fig. S11.jpg]

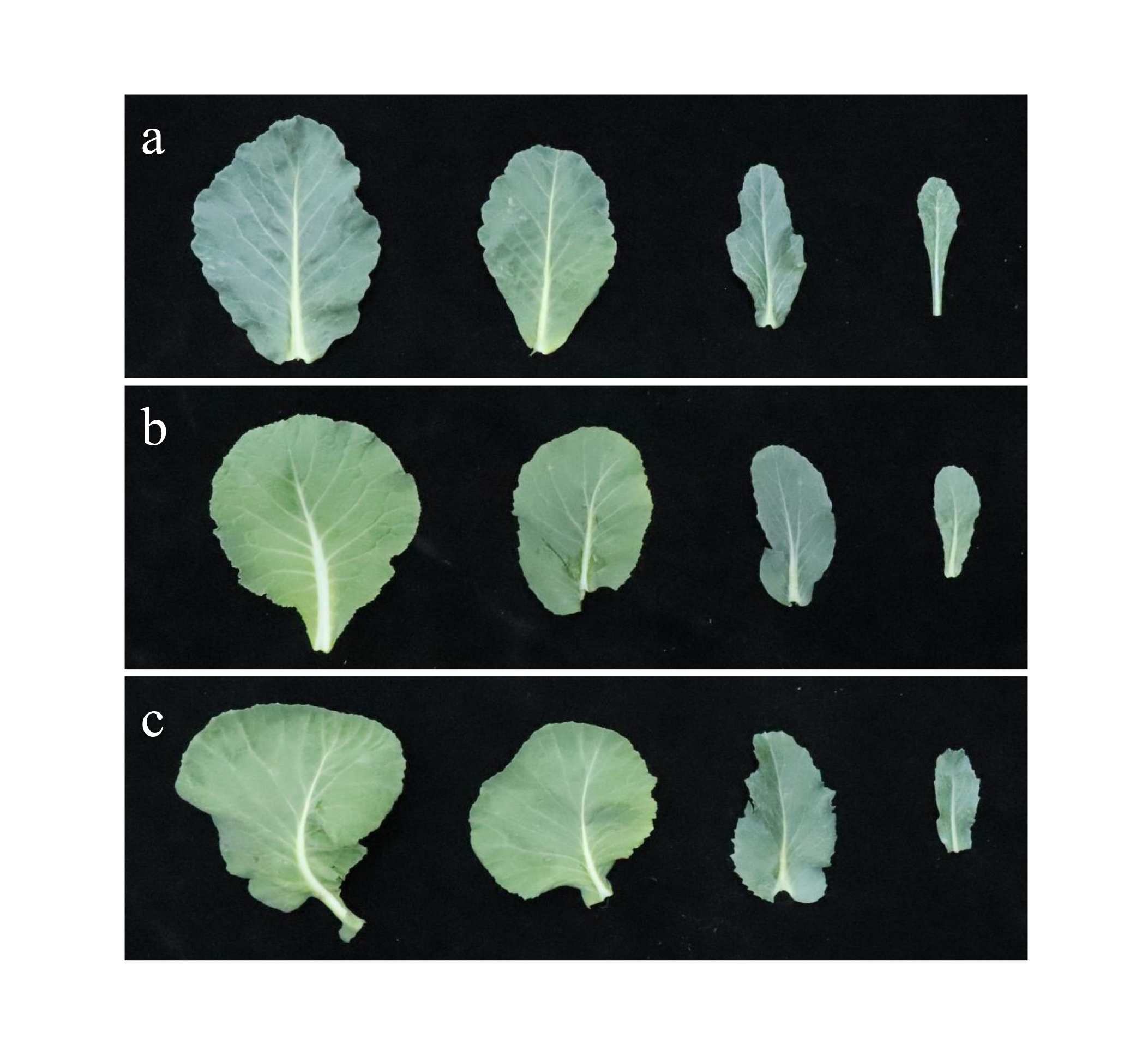

Supplement: Web_Material_uhac195 [file web_material_uhac195.zip › Fig. S12.jpg]

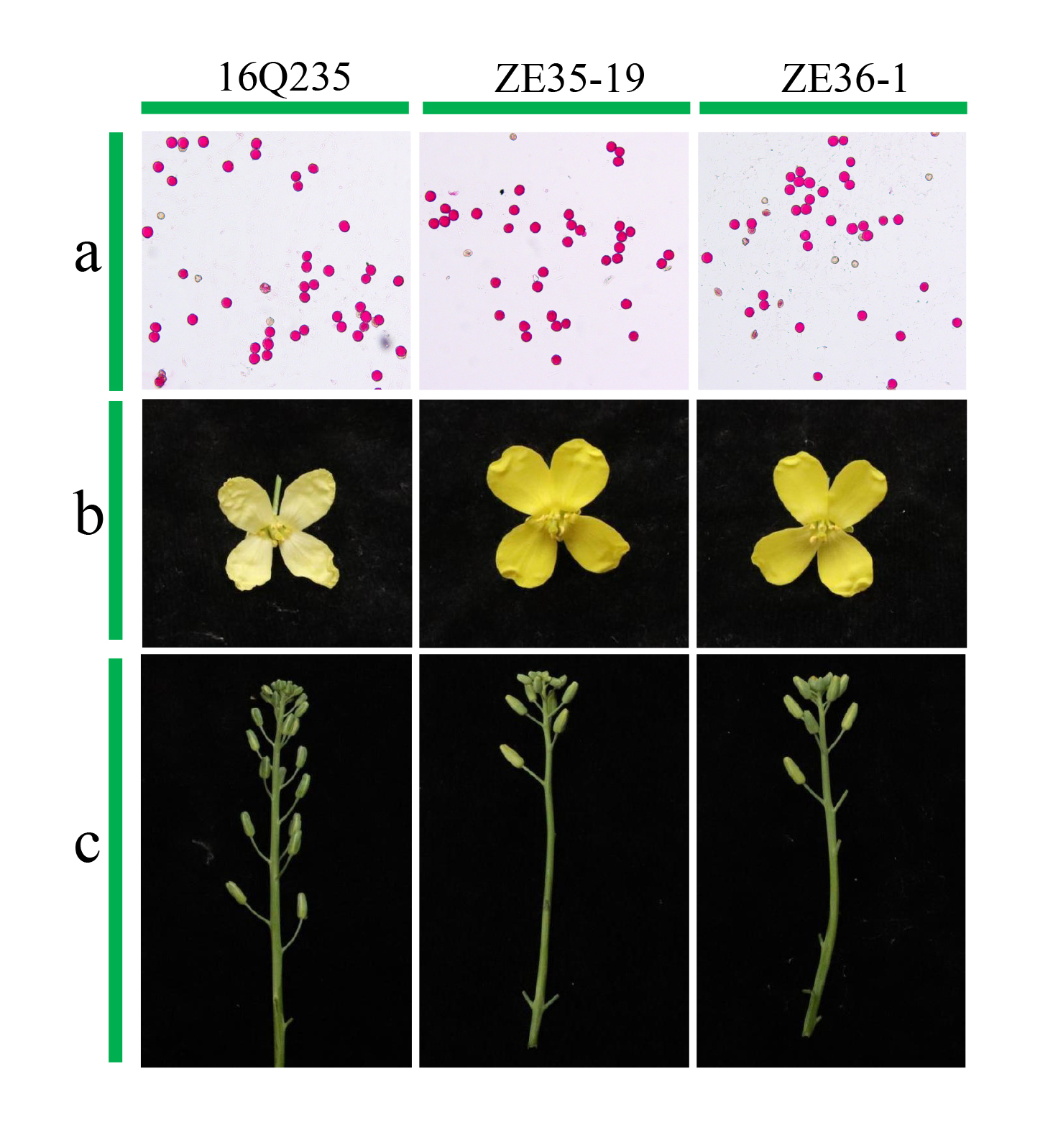

Supplement: Web_Material_uhac195 [file web_material_uhac195.zip › Fig. S13.jpg]

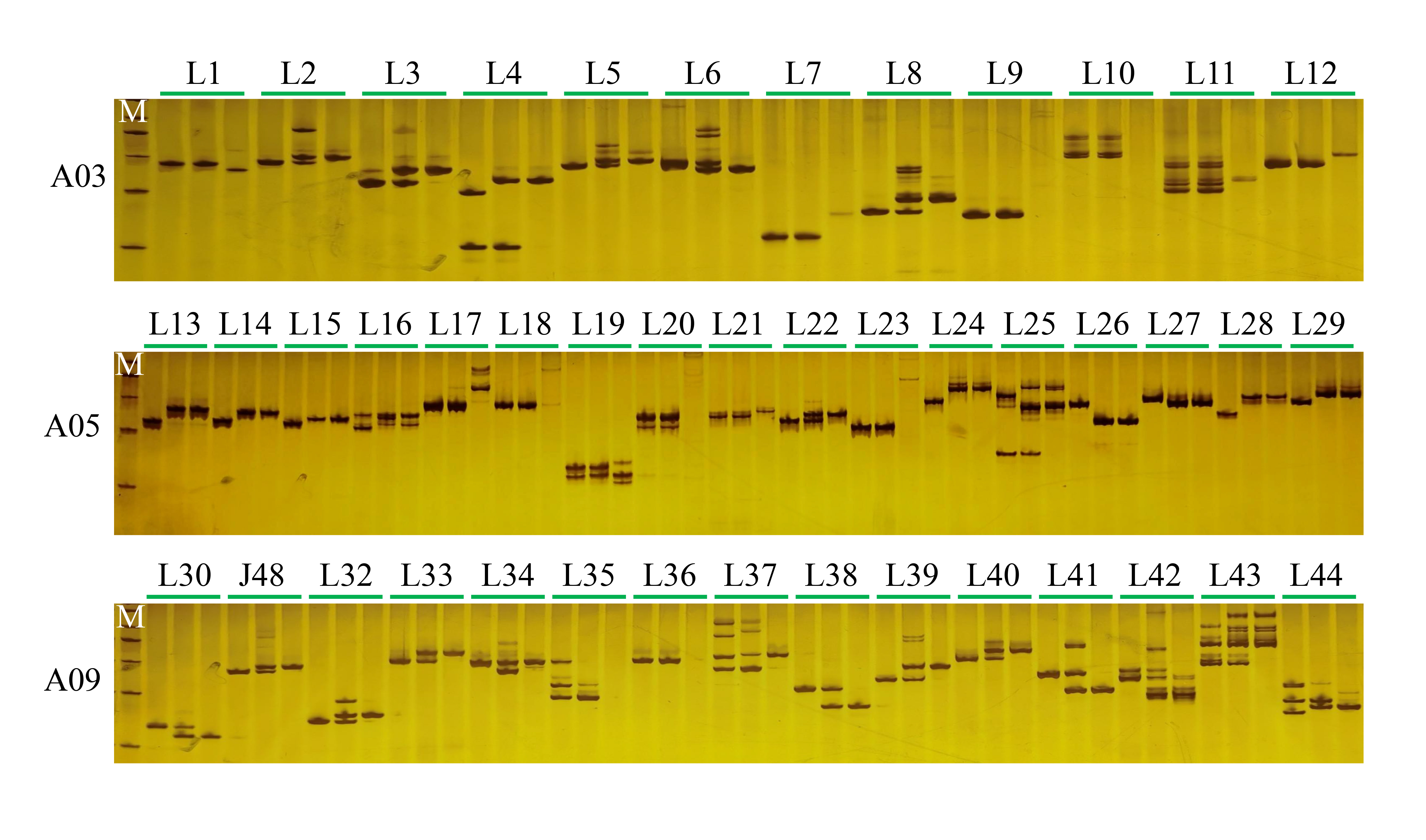

Supplement: Web_Material_uhac195 [file web_material_uhac195.zip › Fig. S14.jpg]

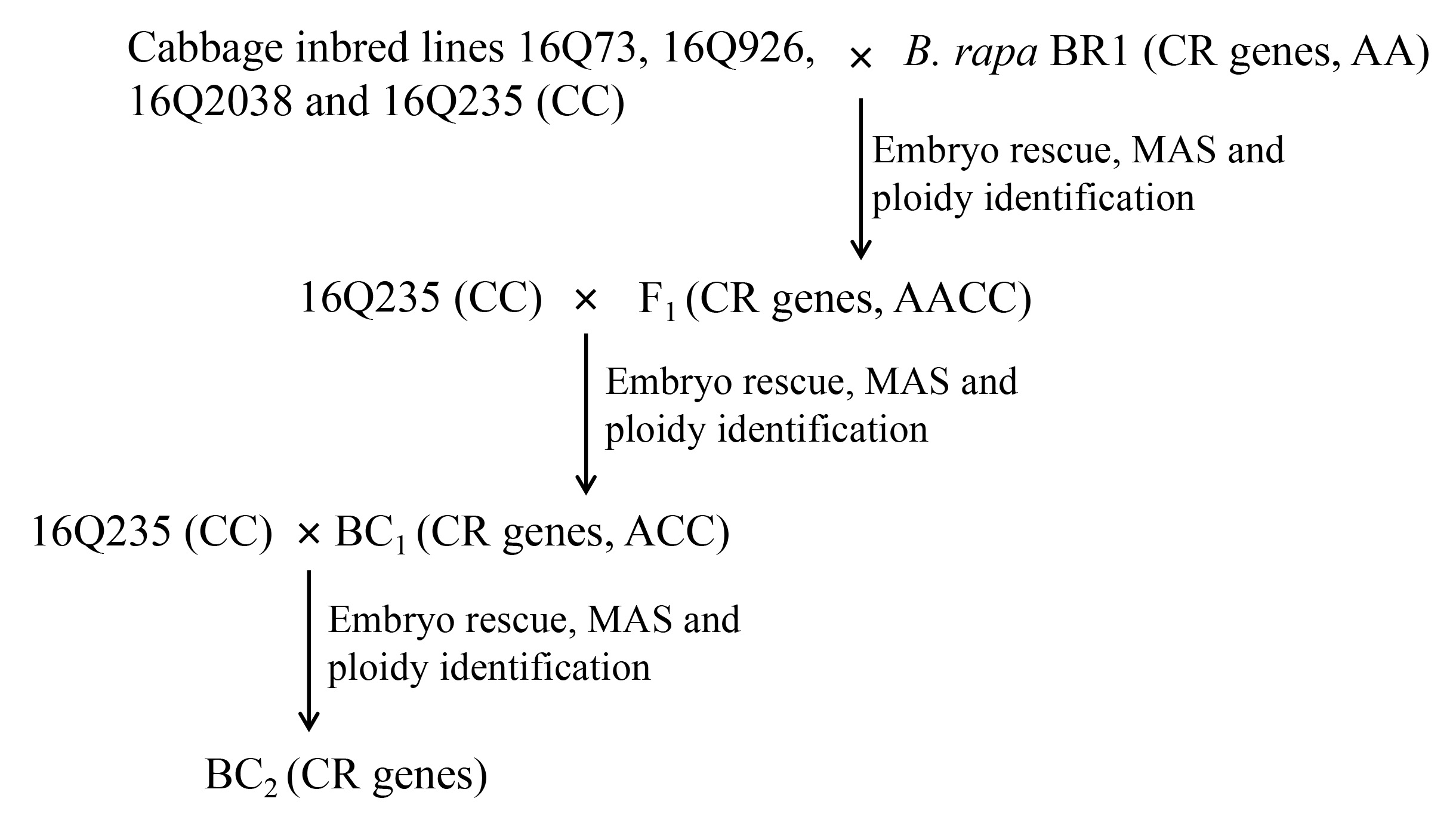

Supplement: Web_Material_uhac195 [file web_material_uhac195.zip › Fig. S15.jpg]

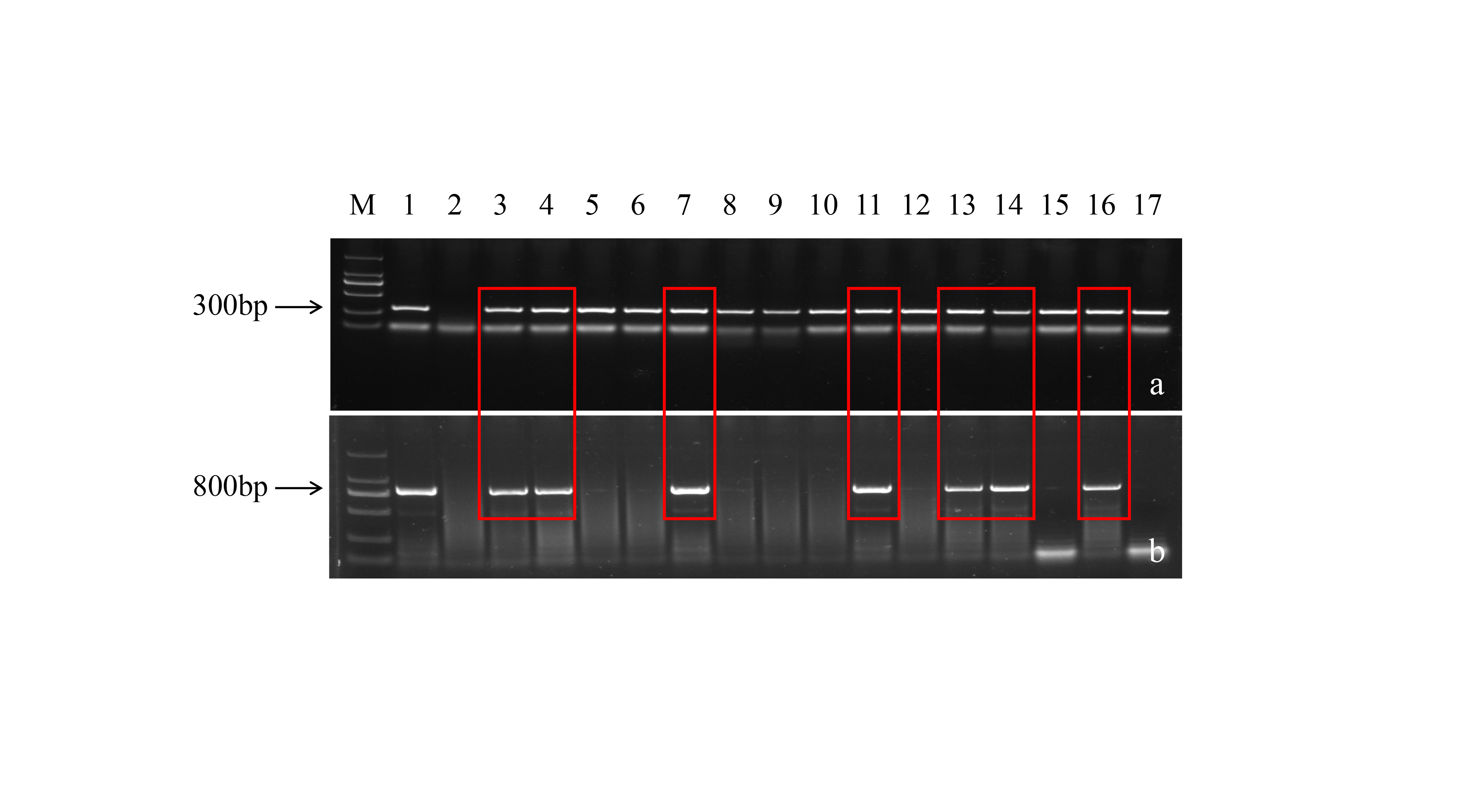

Supplement: Web_Material_uhac195 [file web_material_uhac195.zip › Fig. S2.jpg]

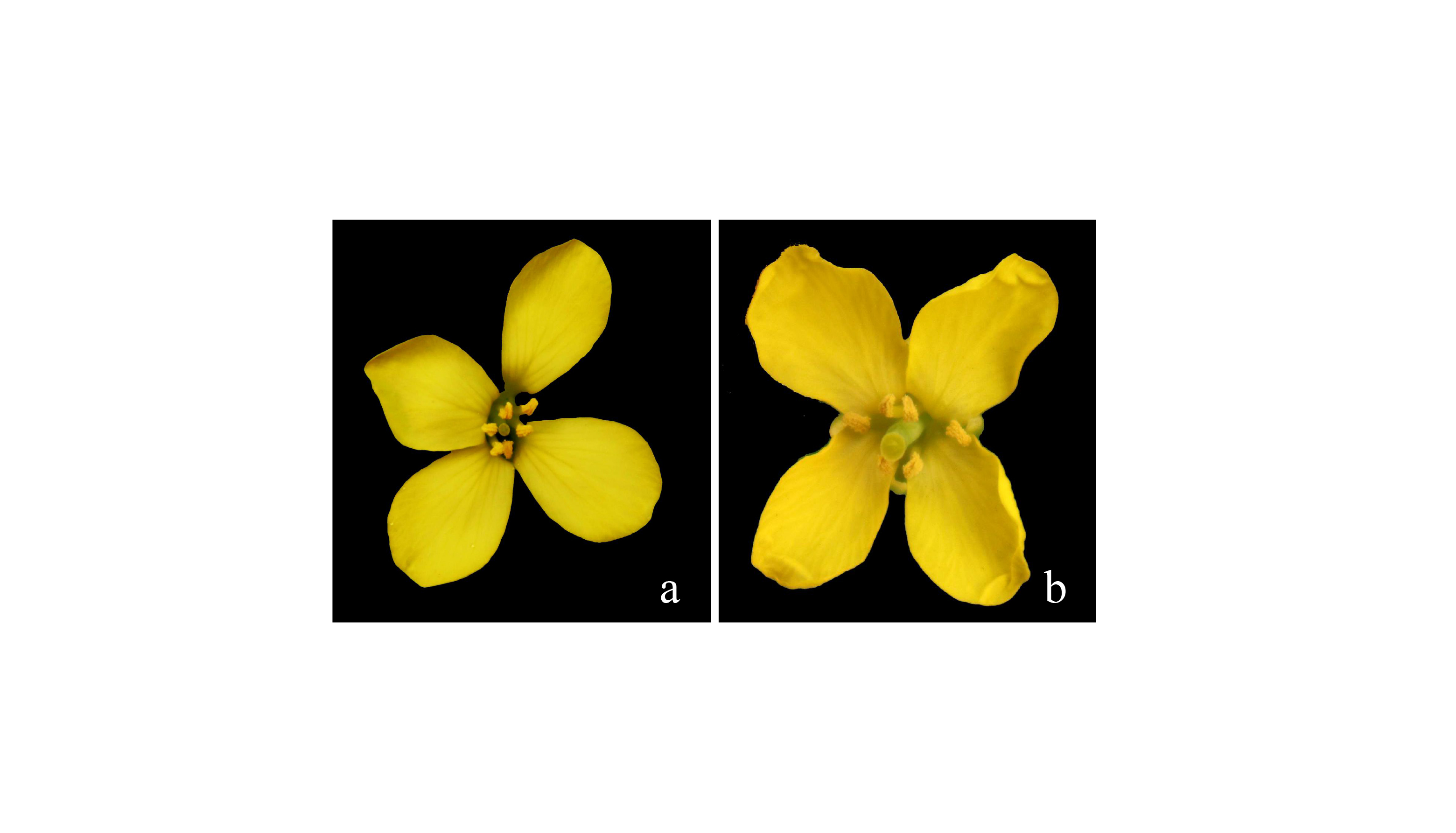

Supplement: Web_Material_uhac195 [file web_material_uhac195.zip › Fig. S3.jpg]

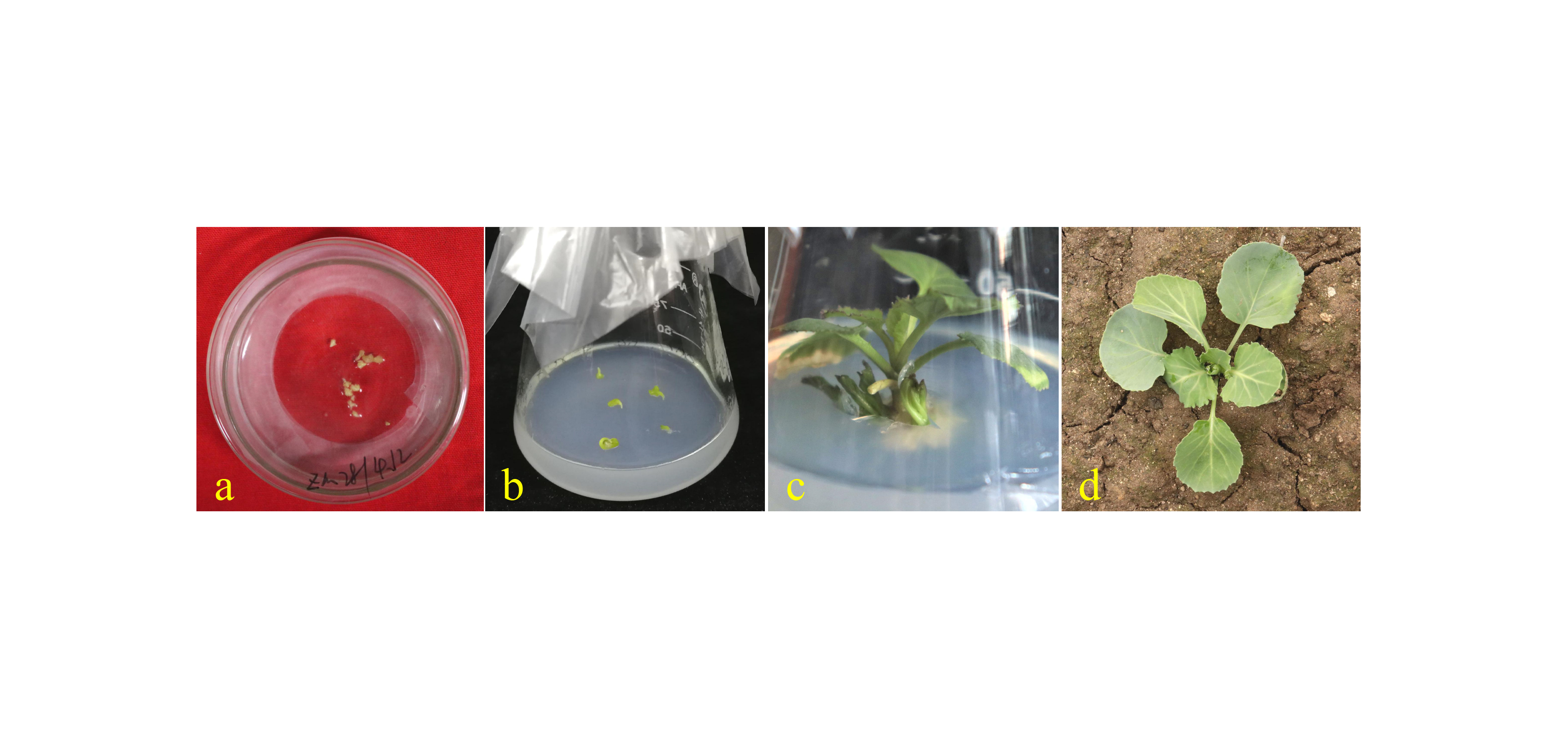

Supplement: Web_Material_uhac195 [file web_material_uhac195.zip › Fig. S4.jpg]

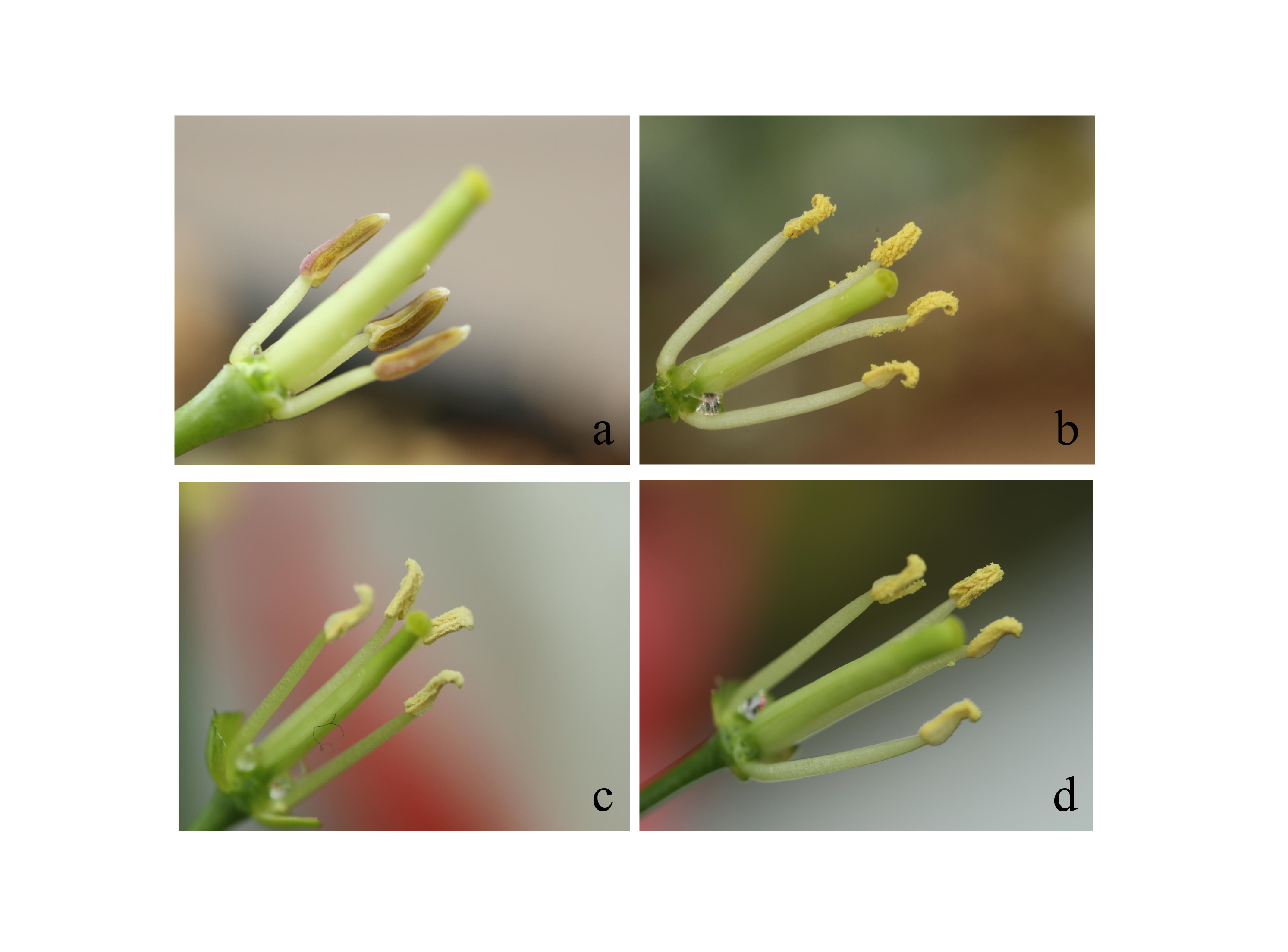

Supplement: Web_Material_uhac195 [file web_material_uhac195.zip › Fig. S5.jpg]

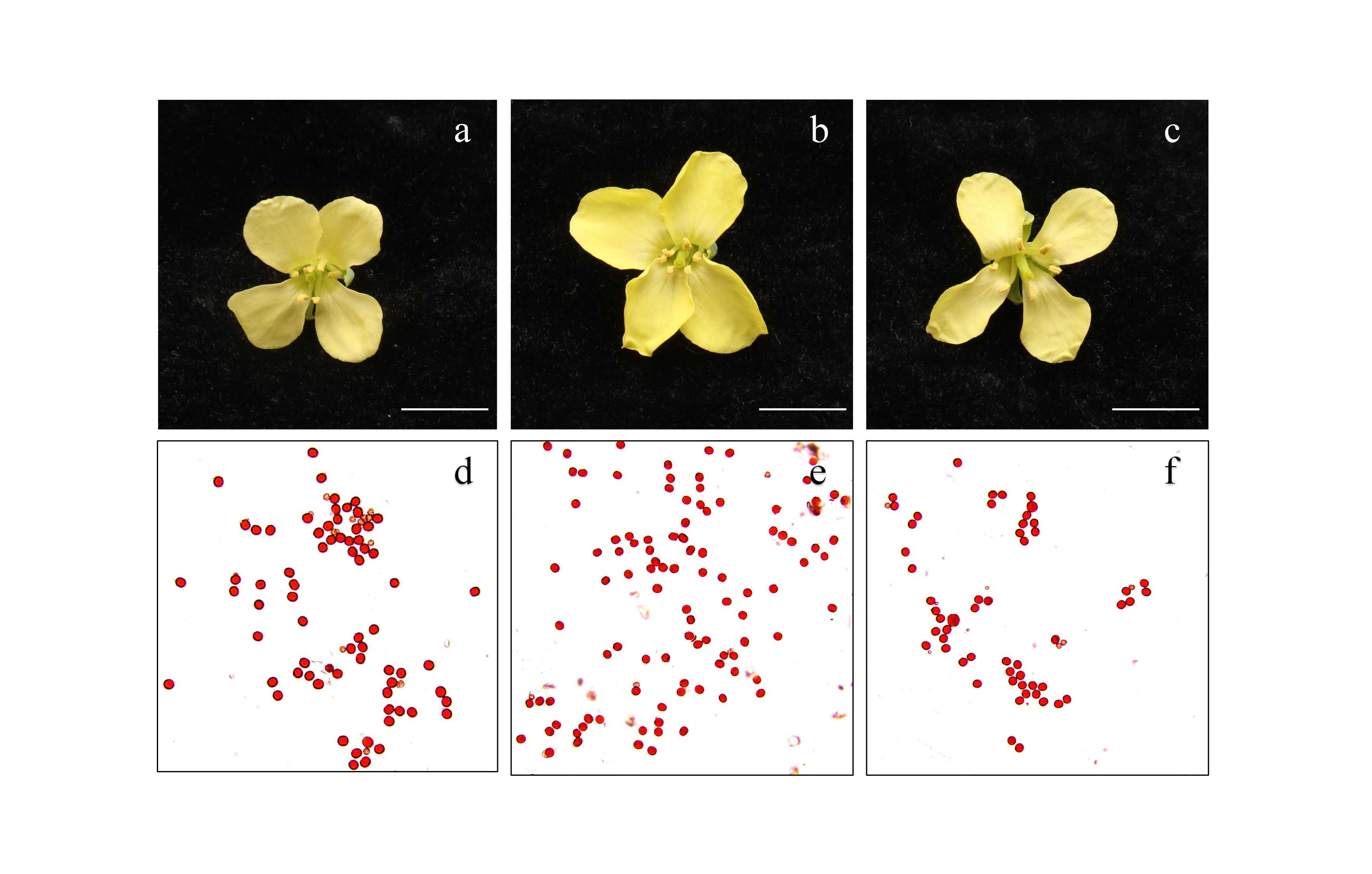

Supplement: Web_Material_uhac195 [file web_material_uhac195.zip › Fig. S6.jpg]

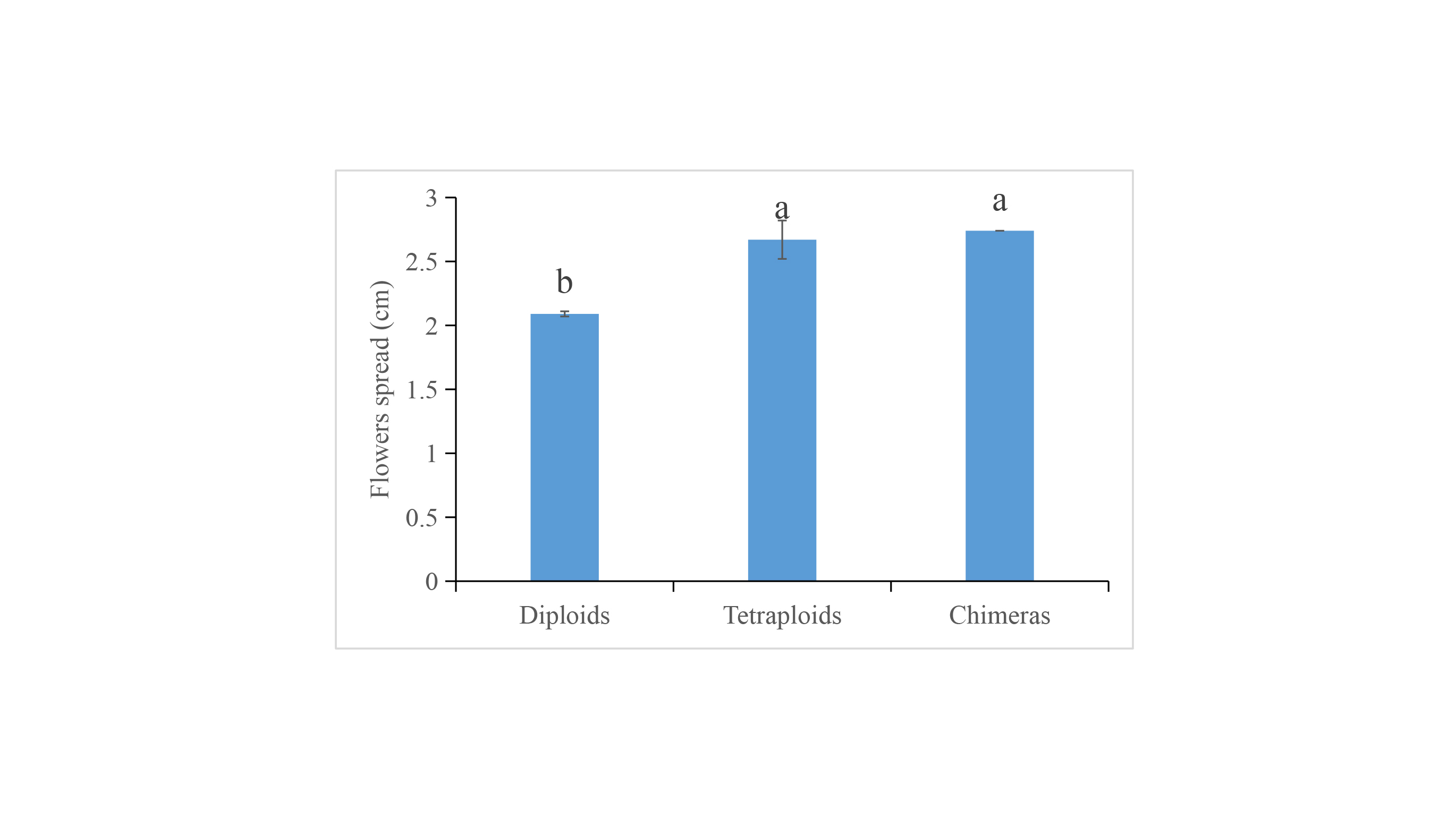

Supplement: Web_Material_uhac195 [file web_material_uhac195.zip › Fig. S7.jpg]

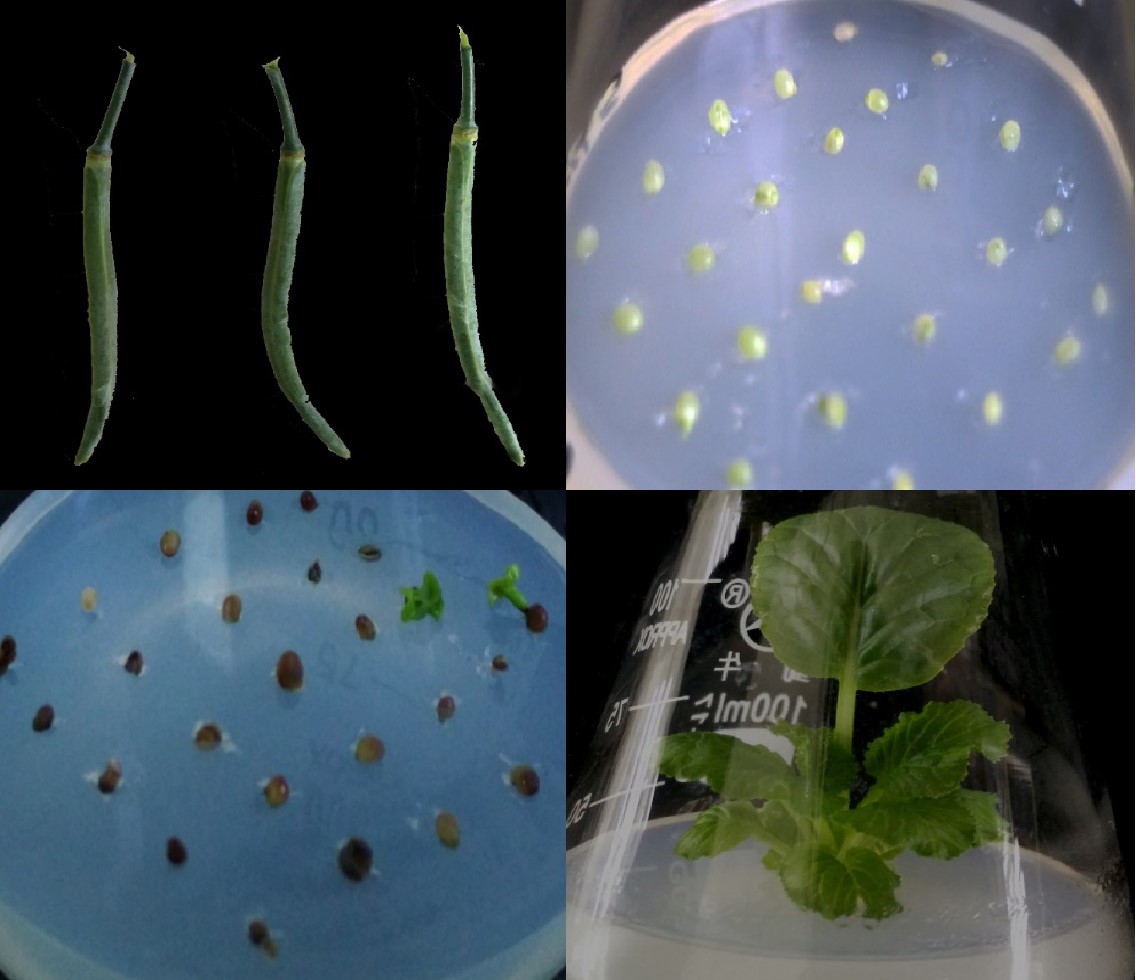

Supplement: Web_Material_uhac195 [file web_material_uhac195.zip › Fig. S8.jpg]

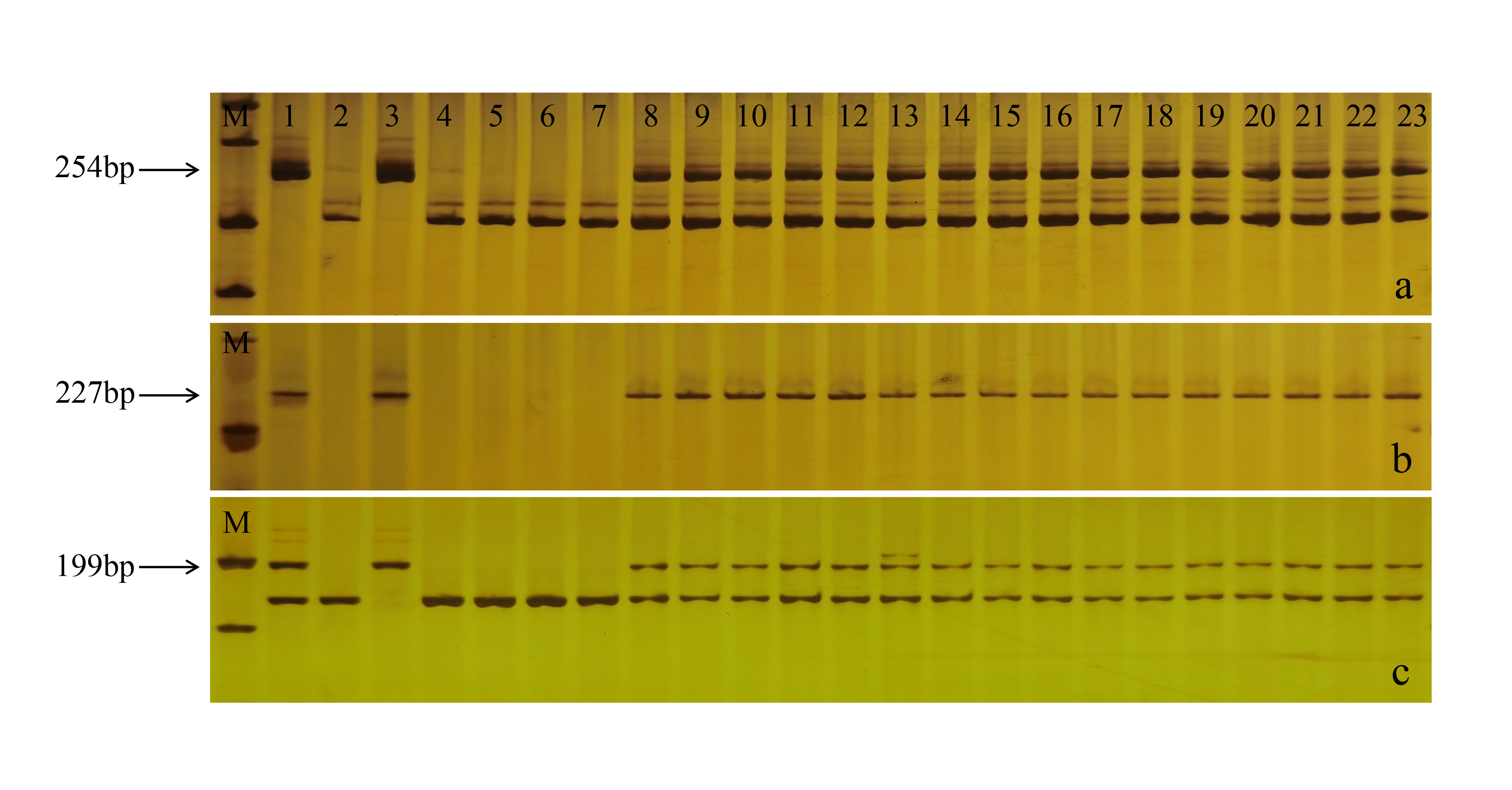

Supplement: Web_Material_uhac195 [file web_material_uhac195.zip › Fig. S9.jpg]
